# Induced clustering of SHP2-depleted tumor cells in vascular islands restores sensitivity to MEK/ERK inhibition

**DOI:** 10.1172/JCI181609

**Published:** 2025-03-25

**Authors:** Yuyi Wang, Hidetaka Ohnuki, Andy D. Tran, Dunrui Wang, Taekyu Ha, Jing-Xin Feng, Minji Sim, Raymond Barnhill, Claire Lugassy, Michael R. Sargen, Emanuel Salazar-Cavazos, Michael Kruhlak, Giovanna Tosato

**Affiliations:** 1 Laboratory of Cellular Oncology, Center for Cancer Research (CCR), National Cancer Institute (NCI), NIH, and; 2 Center for Cancer Research Microscopy Core, Laboratory of Cancer Biology and Genetics, NCI, NIH, Bethesda, Maryland, USA.; 3 Department of Translational Research, Institut Curie, Paris, France.; 4 Division of Cancer Epidemiology and Genetics, NCI, NIH, Rockville, Maryland, USA.; 5 Laboratory of Integrative Cancer Immunology, Center for Cancer Research, NIH, Bethesda, Maryland, USA.

**Keywords:** Angiogenesis, Oncology, Cancer, Chemokines, Endothelial cells

## Abstract

Allosteric inhibitors of the tyrosine phosphatase Src homology 2 domain–containing protein tyrosine phosphatase 2 (SHP2) hold therapeutic promise in cancers with overactive RAS/ERK signaling, but adaptive resistance to SHP2 inhibitors may limit benefits. Here, we utilized tumor cells that proliferate similarly with or without endogenous SHP2 to explore means to overcome this growth independence from SHP2. We found that SHP2 depletion profoundly altered the output of vascular regulators, cytokines, chemokines, and other factors from SHP2 growth-resistant cancer cells. Tumors derived from inoculation of SHP2-depleted, but SHP2 growth–independent, mouse melanoma and colon carcinoma cell lines displayed a typically subverted architecture, in which proliferative tumor cells surrounding a remodeled vessel formed “vascular islands”, each limited by surrounding hypoxic and dead tumor tissue, where inflammatory blood cells were limited. Although vascular islands generally reflect protected sanctuaries for tumor cells, we found that vascular island–resident, highly proliferative, SHP2-depleted tumor cells acquired an increased sensitivity to blockage of MEK/ERK signaling, resulting in reduced tumor growth. Our results show that the response to targeted therapies in resistant tumor cells was controlled by tumor cell–induced vascular changes and tumor architectural reorganization, providing a compelling approach to elicit tumor responses by exploiting tumor- and endothelium-dependent biochemical changes.

## Abstract 2

Induced clustering of tumor cells in vascular islands confers sensitivity to MEK/ERK inhibition in tumor cells resistant to depletion of the SHP2-phosphatase.

## Graphical abstract

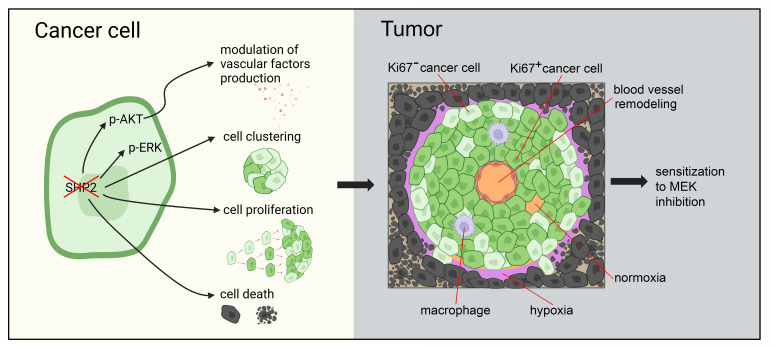

## Introduction

The MAPK pathway, encompassing the RAS/RAF/MEK/ERK signaling components, transmits signals that regulate cell growth from the cell surface to the nucleus (1). In many cancer types, the MAPK signaling pathway is deregulated, resulting in constitutive activation of ERK, which critically contributes to cancer cell growth and survival. The V600E mutation of BRAF, which drives MEK/ERK signaling, is found in approximately 40% of melanomas (2) and approximately 10% of colorectal cancers (3). Despite therapeutic advances, BRAFV600E-mutant metastatic melanoma develops resistance to BRAFV600E inhibitors after initially responding (4, 5), and colorectal cancers are often intrinsically resistant to BRAF kinase inhibitors, underscoring the need to identify potential routes of drug resistance and new targets for these resistant malignancies (6, 7).

The protein tyrosine phosphatase SHP2 is required for full activation of the RAS/ERK pathway, acting upstream of RAS (8, 9). Therefore, SHP2 inhibitors are attractive candidates to control malignancies driven by RAS/ERK signaling, and several of these inhibitors have entered clinical trials. Thus far, resistance to SHP2 inhibitors has been linked to experimental mutations in the protein tyrosine phosphatase (PTP) domain of SHP2, preventing docking of the inhibitor SHP099 into the target, and to other mutations that constrain SHP2 in an open and active conformation (10). Also, genome-wide CRISPR/Cas screens have shown that loss of the *INPPL1*, *MPK5*, or *LZTR1* genes confers resistance to SHP2 inhibitors (7). Additionally, a few tumor lines exhibit intrinsic growth resistance to SHP2 inhibitors (11, 12).

Several observations have highlighted the importance of specialized vascular endothelial cells in the acquisition of resistance to chemotherapy and radiotherapy through various niche-dependent mechanisms (13–16), emphasizing a need to characterize the role of the tumor vasculature in resistance to targeted therapies.

Here, we investigated the contribution of endothelial cells and the tumor vasculature to SHP2 resistance and characterized the biochemical underpinning of cancer-endothelial cell crosstalk. We further reveal a blood vessel–dependent vulnerability of cancer cells, which acquired responsiveness to MEK/ERK targeting.

## Results

### Vascular effects of SHP2 silencing in SHP2-resistant B16F10 melanoma cells.

**Figure 1:**
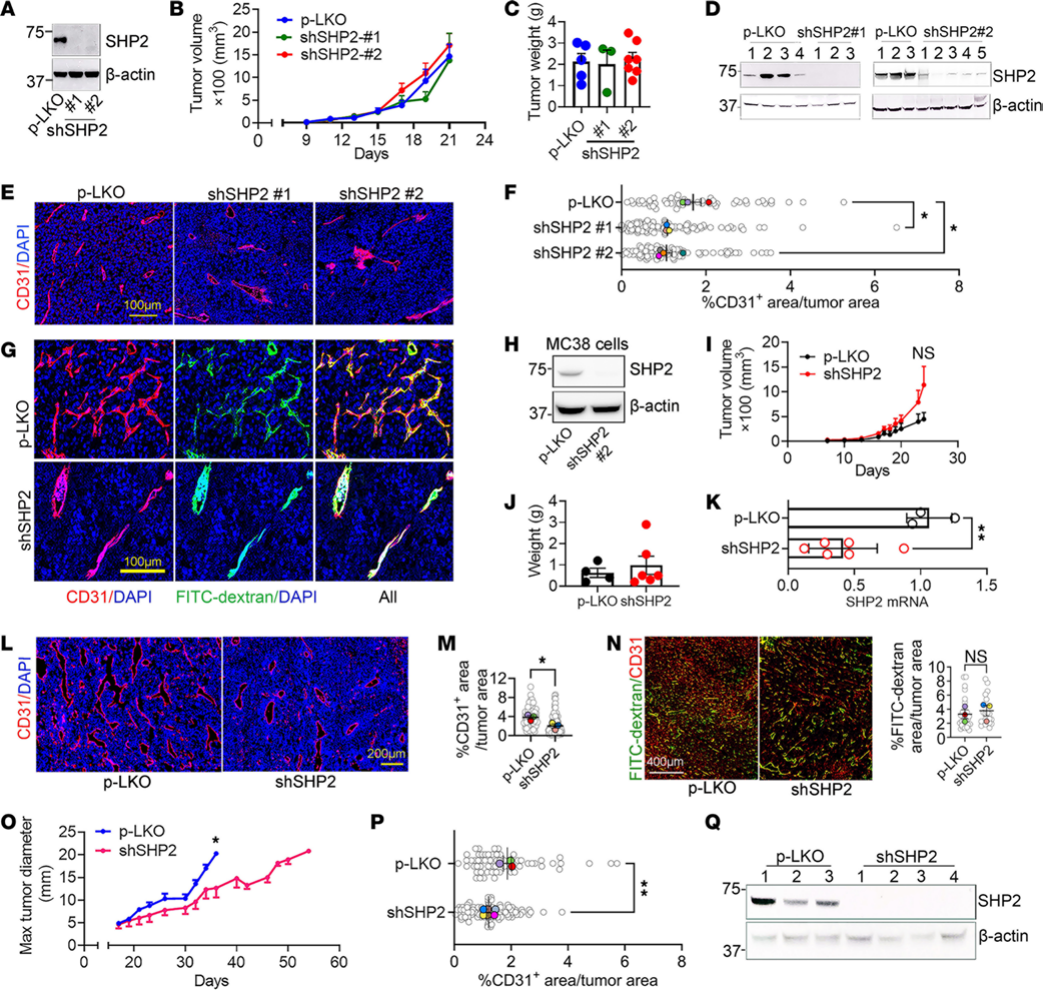
SHP2 depletion in B16F10 cells reduces tumor neovascularization. (**A**) Depletion of SHP2 from B16F10 cells. (**B** and **C**) Tumors from s.c. inoculation of SHP2-silenced and p-LKO-control B16F10 cells (1 × 10^6^). Tumor volume kinetics (**B**) and tumor weight at the endpoint (**C**). Mice: p-LKO, *n* = 6; shSHP2#1 *n* = 7; shSHP2#2, *n* = 7. Data indicate the mean ± SEM. (**D**) SHP2 depletion in SHP2-silenced B16F10 tumors at harvest. (**E** and **F**) Representative images of CD31^+^ vessels in tumors (**E**) and CD31 quantification (**F**). Scale bar: 100 μm. Gray circles: percentage of CD31^+^ area/unit of tumor area; colored dots: tumor mean, p-LKO tumors (*n* = 3); shSHP2 tumors (mL1, *n* = 3; mL2, *n* = 4). *P < 0.05 by 1-way ANOVA. (**G**) Images of vessel perfusion by FITC-dextran; SHP2-silenced (shmL2). Scale bar: 100 μm. (**H**–**K**) p-LKO and SHP2-silenced MC38 colon carcinoma cells (**H**) generated s.c. tumors in mice (p-LKO *n* = 4; shSHP2 *n* = 8) (**I**) with similar tumor weights at harvest (day 24) (**J**). Harvested shSHP2-MC38 tumors expressed less *Shp2* mRNA than did p-LKO control tumors (**K**). **P < 0.01, by 2-tailed Student’s *t* test. (**L** and **M**) SHP2-depleted MC38 tumors (*n* = 3) were less vascularized than p-LKO control tumors (*n* = 3). Representative image (**L**) and quantification (**M**). Scale bar: 200 μm. Gray circles: percentage of CD31^+^ area/unit of tumor area; colored dots: tumor mean. *P < 0.05, by 2-tailed Student’s *t* test. (**N**) Tumor vessel perfusion by FITC-dextran visualization; representative images (left) and FITC quantification (right) in p-LKO control (*n* = 3) and shSHP2-silenced tumors (*n* = 3). Scale bar: 400 μm. Gray circles: percentage of FITC-dextran area/unit of tumor area; colored dots: tumor mean. (**O**) Tumor growth from s.c. inoculation of SHP2-silenced (shSHP2mL1 or mL2; *n* = 8) and p-LKO control (*n* = 3) B16F10 cells (2 × 10^4^). Individual mice were euthanized when the maximal (Max) tumor diameter was/approached 20 mm or at humane endpoints. On day 36: *P < 0.05, by 2-tailed Student’s *t* test. (**P**) Quantification of the percentage of CD31^+^ blood vessels; *n* = 3 mice/p-LKO; *n* = 6 mice/shSHP2 (day 36). Gray circles: percentage of CD31^+^ area/unit of tumor area; colored dots: tumor mean. **P < 0.01, by 2-tailed Student’s *t* test. (**Q**) SHP2 was depleted (day 36) in cells of shSHP2#1- or shSHP2#2-silenced B16F10 tumors.

Previously, we found that the mouse melanoma B16F10 cell line is not dependent on the activity of SHP2 for proliferation in culture, since SHP2 silencing and the irreversible SHP2 inhibitor SHP099 do not inhibit B16F10 proliferation in vitro (11). Also, SHP2-silenced B16F10 tumors grow similarly to control (p-LKO vector) B16F10 tumors in syngeneic mice (11). Thus, B16F10 tumor cells can be considered growth resistant to SHP2 depletion (7). Here, we confirmed that effective and sustained SHP2 reduction by distinct shRNAs did not reduce the growth of B16F10 cells in syngeneic mice (Figure 1, A–D, and Supplemental Figure 1, A–D; supplemental material available online with this article; https://doi.org/10.1172/JCI181609DS1). Unexpectedly, however, we observed that tumor vascularization was significantly reduced in SHP2-silenced B16F10 tumors compared with control tumors (Figure 1, E and F, and Supplemental Figure 1, E and F). FITC-dextran intensity/per unit of tumor area showed that vessels in the SHP2-depleted B16F10 tumors were perfused to a similar degree as those in the controls, which was indicative of increased vessel perfusion in the less vascularized SHP2-silenced tumors compared with p-LKO tumors (Figure 1G and Supplemental Figure 1, G and H). H&E staining showed similarly viable tumor tissue in the control and SHP2-silenced tumors (Supplemental Figure 1, I and J). Also, the proportion of lungs with microscopic metastases (generally 1 or 2 per lung) was similar in the control and SHP2-silenced tumors (Supplemental Figure 1K).

The mouse colon carcinoma MC38 and CT26 cell lines are also growth resistant to SHP2 inhibition (11, 12). We found that tumors generated by inoculation of SHP2-silenced MC38 (Figure 1H) and CT26 (Supplemental Figure 1L) cell lines resembled those induced by SHP2-silenced B16F10 melanoma, as they had similar growth compared with the nonsilenced (p-LKO) control tumors (Figure 1, I–K, and Supplemental Figure 1, M and N), reduced vascularization compared with controls (Figure 1, L and M, and Supplemental Figure 1, O and P), and similar vessel perfusion per unit of tumor area compared with control tumors (Figure 1N).

The reduced vascularization of tumors derived from inoculation of SHP2-silenced cell lines, which are themselves growth–insensitive to SHP2 depletion, was unexpectedly uncoupled from reduced tumor growth at endpoint (Figure 1, B, C, I, and J, and Supplemental Figure 1, B and M). We examined whether extending tumor observation might show an antitumor effect potentially from reduced vascularization of SHP2-silenced tumors. Thus, we injected fewer B16F10 tumor cells (2 × 10^4^, Figure 1O, and 2.5 × 10^4^ Supplemental Figure 1Q) and euthanized the mice individually, when the maximum tumor diameter reached or approached a diameter of 20 mm in any direction. In these experiments, tumors generated by injection of SHP2-silenced B16F10 cells eventually grew at a somewhat reduced rate compared with controls, reaching the endpoint (~20 mm in maximal diameter) later than did the control tumors (Figure 1O and Supplemental Figure 1Q). Delayed SHP2-silenced B16F10 tumor growth was associated with reduced vascularization compared with controls (Figure 1P). These results show that SHP2-depleted tumor cells (Figure 1Q), whose growth was independent of SHP2 in vitro, generated tumors that were less vascularized and less proliferative than controls, potentially due to SHP2 regulation of signaling pathways other than RAS/ERK signaling.

### Effects of SHP2 silencing on PI3K, MAPK, and STAT signaling in B16F10 tumor cells.

Since the SHP2 phosphatase is required for full activation of the RAS/ERK signaling pathway but also has context-dependent effects on other signaling pathways (8, 9, 17, 18), we compared key signaling pathways in B16F10 cells from culture and from B16F10 tumors.

**Figure 2:**
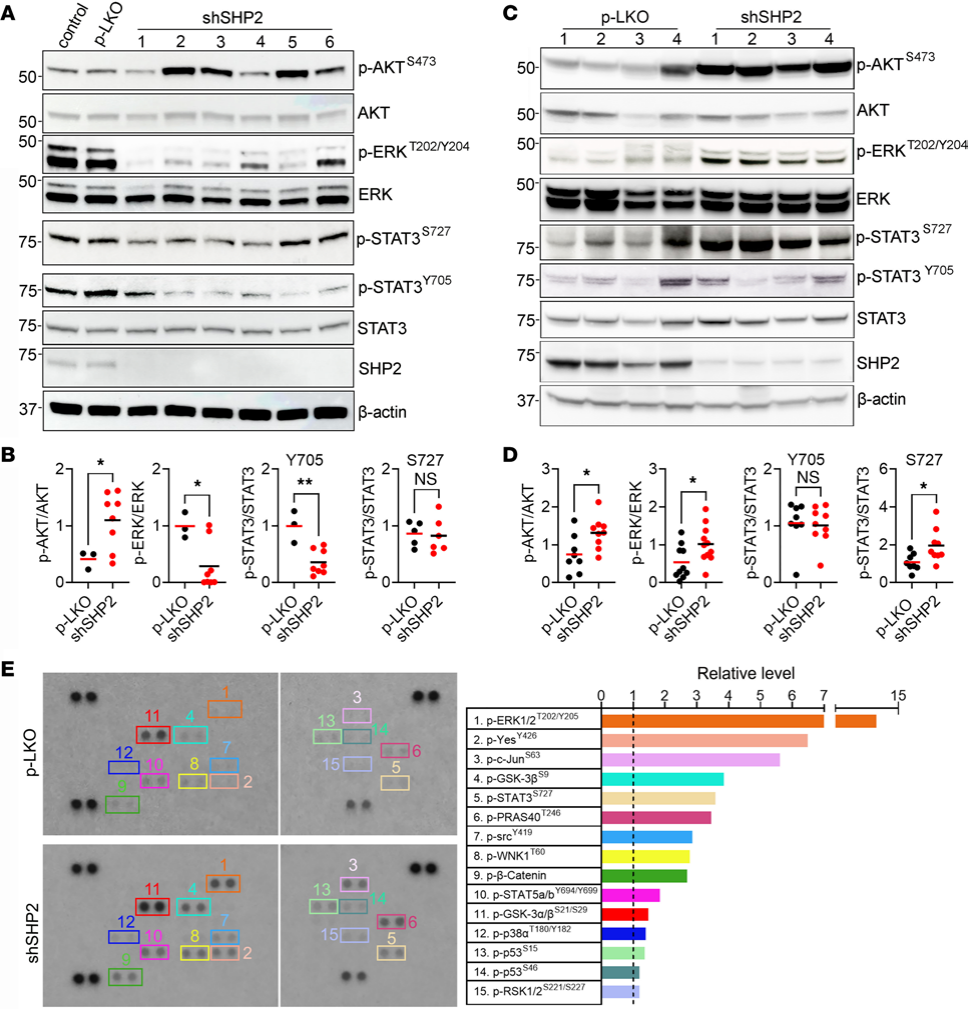
Distinct signaling profiles of SHP2-depleted B16F10 cells from culture or mice. (**A** and **B**) Activity of signaling molecules in B16F10 cells propagated in culture after SHP2 silencing with 6 different shRNAs. Representative immunoblots (**A**) and quantification (**B**) of relative band intensities in control and SHP2-silenced cells. (**C** and **D**) Lysates from SHP2-silenced and p-LKO control B16F10 tumors harvested on day 21 after cell inoculation were evaluated by immunoblotting. Representative immunoblot results from individual tumors (**C**) and band quantification (**D**). (**E**) Signaling profile of SHP2-silenced and control B16F10 tumors harvested on day 21 after inoculation evaluated with a phospho-kinase assay kit. Lysates (900 mg) from pools of 4 p-LKO and 4 SHP2-silenced tumors were applied to the array. Visualization of the results and quantification of the relative pixel density in p-LKO control and SHP2-silenced tumor lysates are shown. The quantitative results are expressed relative to the control (identified by the dotted line). *P < 0.05 and **P < 0.01, by 2-tailed Student’s *t* test. (**B** and **D**) The horizontal lines reflect the mean.

Compared with p-LKO control cells, the levels of phosphorylated ERK1/2^T202/Y204^ (p-ERK1/2^T202/Y204^) and p-STAT3^Y705^ were generally reduced in SHP2-silenced B16F10 cells from in vitro culture, whereas the levels of p-Akt^S473^ were generally increased and the levels of p-STAT3^S727^ were similar (Figure 2, A and B, and Supplemental Figure 2A). B16F10 tumor lysates (composed mostly of tumor cells) showed that p-Akt^S473^ levels were increased in the SHP2-silenced tumors compared with levels in control tumors (Figure 2, C and D), a change that was similar to that observed in SHP2-silenced B16F10 cells from culture. However, unlike in B16F10 cells propagated in vitro, we found that p-ERK1/2^T202/Y204^ levels were increased (Figure 2, C and D, and Supplemental Figure 2B). Also, p-STAT3^S727^ levels were generally increased in SHP2-silenced B16F10 tumors compared with controls, whereas p-STAT3^Y705^ levels were similar in the 2 groups (Figure 2, C and D). The notable difference in p-ERK1/2^T202/Y204^ (reduced in SHP2-silenced B16F10 cells from culture and increased in SHP2-silenced B16F10 tumors) could not be attributed simply to the timing difference from SHP2 silencing since p-ERK1/2^T202/Y204^ continued to be reduced in B16F10 cells cultured for 16 weeks after SHP2 silencing (Supplemental Figure 2C).

A phospho-kinase array kit largely confirmed and extended the signaling profile of B16F10 tumors characterized by immunoblotting (Figure 2E). In addition to detecting increased activity of p-ERK1/2^T202/Y204^ in SHP2-silenced B16F10 tumors compared with controls, the phospho-kinase array showed increased activity of p–c-Jun^S63^ and p–GSK-3b^S9,S21/S29^, which are mediators of a signaling pathway linking active ERK with increased JNK/c-Jun phosphorylation signaling via stabilization by GSK-3 that is active in progressive melanoma (19). The array also showed that the Src kinase family member p-Yes^Y426^ and, to a lesser degree, p-Src^Y419^, as well as the targets of p-AKT, p-PRAS40^T246^, and p-WNK1^T60^ proteins, were more active in SHP2-silenced B16F10 tumors than in control tumors, consistent with the known relative roles of these kinases in melanoma and cancer progression (20–22). Confirming the immunoblotting results, the array also detected increased p-STAT3^S727^ levels.

**Figure 3:**
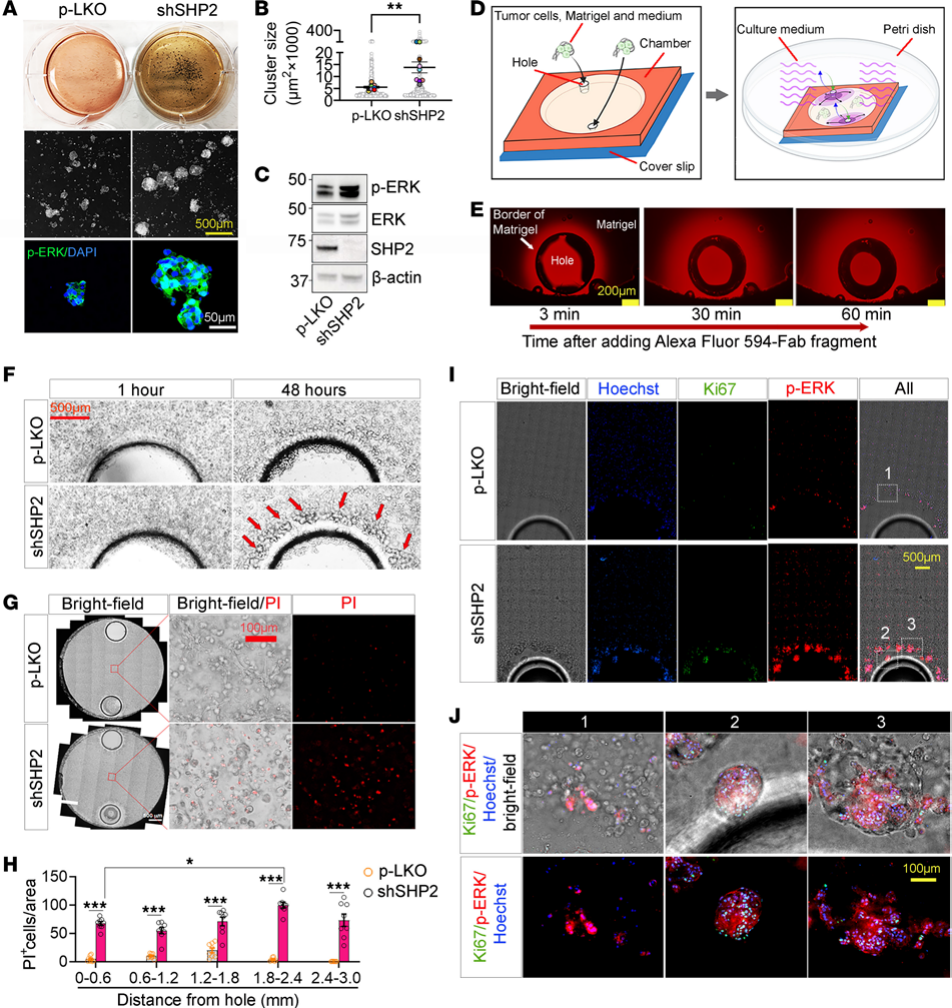
SHP2 silencing regulates B16F10 cell clustering, ERK activation, cell proliferation, and cell death in 3D culture models. (**A**) Control p-LKO and SHP2-silenced B16F10 cells cultured (14 days) in ultra-low attachment plates were visualized by bright-field imaging (top and mid panels show representative images). Confocal cluster images are shown in the bottom panels (p-ERK, green; DAPI/nuclei, blue). Scale bars: 500 μm (middle panel) and 50 μm (bottom panel). (**B**) Quantification of cell cluster size; gray circles reflect individual clusters; colored dots reflect the mean cluster size/culture (*n* = 9); results are presented as mean (horizontal lines). **P < 0.01 by 2-way Student’s *t* test. (**C**) Immunoblot results of p-ERK^T202/Y204^ in lysates of B16F10 cells cultured for 7 days on ultra-low attachment plates. (**D**) Schematic diagram illustrating the experimental in vitro culturing system; left: a suspension of tumor cells, Matrigel, and DMEM was introduced into a tight-sealed chamber to evenly occupy the space; right: the chamber was transferred into a Petri dish containing DMEM with 10% FBS, which entered the chamber only through the 2 holes. (**E**) Fluorescence images of chamber holes and surrounding Matrigel 3, 30, and 60 minutes after addition of the Alexa Fluor 594–Fab fragment, showing the time-dependent fluorescence diffusion from the hole. Scale bar: 200 μm. (**F**) Representative bright-field images of control and SHP2-silenced B16F10 cells surrounding the chamber holes after 1- and 48-hour incubations (*n* = 3 experiments). The red arrows point to cell clustering around the hole. Scale bar: 500 μm. (**G**) Representative bright-field and fluorescence images of the indicated areas after staining with PI (48-hour incubation; *n* = 3 experiments). Scale bar: 500 μm (left panels) and 100 μm (middle and right panels). (**H**) Number of PI^+^/dead p-LKO and SHP2-silenced B16F10 cells as a function of distance from the corresponding hole (*n* = 3 experiments). ***P < 0.001, by 2-tailed Student’s *t* test. Results are presented as means± SEM. (**I**) Representative images of chambers containing B16F10 cells stained with Hoechst (nuclei/blue), Ki67 (green), and p-ERK (red) (48-hour incubation; *n* = 3 experiments). Scale bar: 500 μm. (**J**) Magnified images of the areas outlined in **I** (area 1: p-LKO B16F10 cells; areas 2 and 3: SHP2-silenced B16F10 cells) showing the cell clusters. Scale bar: 100 μm.

These results provide evidence that SHP2 silencing activated AKT in B16F10 cells from culture and from tumors. In contrast, SHP2 silencing reduced p-ERK in B16F10 cells propagated in vitro and increased p-ERK levels in SHP2-silenced B16F10 cells propagated in vivo as tumors. To dissect the role of SHP2 silencing in modulating p-ERK levels in B16F10 cells in vitro and in vivo, we altered the culture conditions for B16F10 cells to model aspects of B16F10 cells propagated as tumors. First, we cultured the cells on ultra-low attachment plates to generate 3D cell clusters (23). Under these conditions, SHP2-silenced B16F10 cells formed significantly larger-sized and darker (from melanin accumulation) clusters compared with control B16F10 cells (Figure 3, A and B). In these clusters, the SHP2-silenced B16F10 cells contained more p-ERK than did control B16F10 cells (Figure 3, A and C).

In another in vitro system (schematically shown in Figure 3D), we suspended B16F10 cells into Matrigel/DMEM medium (without serum) and placed the mixture into sealed, impermeable chambers. These chambers are designed with 2 holes on opposite sides to allow the supply of complete culture medium (with serum) and oxygen. This setup created a nutrient gradient emanating from the holes (Figure 3E). After a 48-hour incubation, SHP2-silenced B16F10 cells reproducibly clustered, proliferated (Ki67^+^), and expressed p-ERK around the holes/source of nutrients and oxygen, whereas p-LKO B16F10 cells minimally clustered and rarely stained for Ki67 or p-ERK (Figure 3, F–J). Instead, p-LKO B16F10 cells maintained a similar distribution within the chamber (bright-field imaging, Figure 3F), and, in areas distant from the ports, these cells were less viable than SHP2-silenced B16F10 cells, as detected by propidium iodide (PI) staining (Figure 3, G and H). These experiments in vitro underline the critical importance of SHP2 silencing in controlling ERK activation, cell clustering, proliferation, and death in B16F10 cells under distinct 3D culture conditions.

### Phenotypic differences between SHP2-depleted and SHP2-not depleted B16F10 tumors.

Next, we analyzed key tumor markers in sections of tumors from SHP2-silenced and control B16F10 cells.

**Figure 4:**
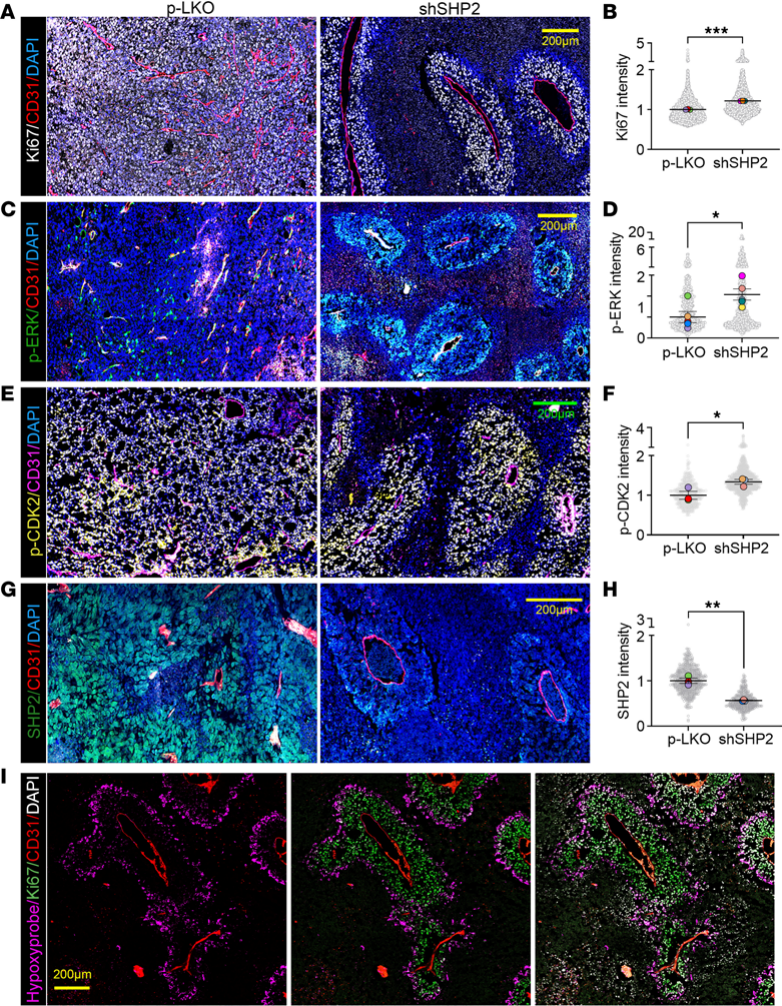
Characterization of SHP2-silenced B16F10 tumor architecture. (**A** and **B**) Ki67 and CD31 immunostaining identified a distinct structural organization of SHP2-silenced B16F10 tumors. Nuclei are stained with DAPI. Representative confocal microscopy images (**A**) and quantification (**B**) of Ki67 fluorescence intensity in tumor vascular islands from SHP2-silenced tumors (*n* = 3) and p-LKO control tumors (*n* = 3). (**C** and **D**) p-ERK^T202/Y205^ and CD31 immunostaining detected p-ERK in representative tumor vascular islands of SHP2-silenced B16F10 tumors (**C**). Quantification of p-ERK fluorescence in live tissue from p-LKO tumors (*n* = 5) and islands of SHP2-silenced tumors (*n* = 5) (**D**). (**E** and **F**) p-CDK2^T160^ and CD31 immunostaining identified the distinct structural organization of SHP2-silenced B16F10 tumors (**E**). p-CDK2 fluorescence quantification in p-LKO (*n* = 3) and SHP2-silenced (*n* = 3) tumor islands (**F**). (**G** and **H**) SHP2 and CD31 immunostaining detected strong SHP2 fluorescence intensity in representative tumor islands of control B16F10 tumors and faint SHP2 fluorescence in SHP2-silenced B16F10 tumor islands (**G**). SHP2 quantification in live tissue from p-LKO (*n* = 3) and SHP2-silenced (*n* = 3) tumor islands (**H**). In **B**, **D**, **F**, and **H**, the gray circles reflect fluorescence intensity/unit of tumor area; colored dots reflect the mean fluorescence intensity/tumor analyzed. (**I**) Representative confocal images of tumor islands from SHP2-silenced B16F10 tumors displaying the central CD31^+^ vessel surrounded by Ki67^+^ tumor cells limited at the periphery by a hypoxic zone (Hypoxyprobe^+^) and further surrounded by areas lacking identifiable DAPI^+^ cells. In **B**, **D**, **F**, and **H**, horizontal lines indicate the mean ± SEM. *P < 0.05, **P < 0.01, and ***P < 0.001, by 2-tailed Student’s *t* test. Scale bars: 200 μm.

Imaging to detect Ki67 showed that SHP2-silenced tumors acquired a distinctive structural organization, consisting of “islands” of proliferating (Ki67^+^) tumor cells surrounding a central blood vessel, separated from each other by nonproliferating (Ki67^–^) tumor tissue mostly devoid of blood vessels (Figure 4A and Supplemental Figure 3, A and B). These Ki67^–^ areas comprised mostly dead or dying (cleaved caspase 3^+^) tumor tissue (Supplemental Figure 3C). Quantitatively, the Ki67 staining intensity was significantly greater in the tumor islands within SHP2-silenced B16F10 tumors than in control B16F10 tumors (Figure 4B). The viable blood vessels of SHP2-silenced B16F10 tumors differed morphologically from those in the control tumors, as the former showed reduced branching complexity (Figure 4A) accompanied by an increase in vessel caliber (Supplemental Figure 3, D and E), thus resembling tumor vessels in mice treated with agents that prune “immature” tumor vessels and “normalize” the remaining tumor vessels (24–26). Further characterization of the vasculature in SHP2-silenced B16F10 tumors showed that the vessels had more abundant coverage with collagen IV, laminin, and NG2^+^ pericytes than did the vessels in the control tumors (Supplemental Figure 4, A–F). Also, the proportion of Ki67^+^ and Ki67^+^p-CDK2^T160^^+^ endothelial cells (CD31^+^) was reduced in the SHP2-silenced B16F10 tumors compared with control tumors (Supplemental Figure 4, G–I), indicating greater endothelial cell quiescence in SHP2-silenced tumors. Functionally, the vessels in SHP2-silenced B16F10 tumors allowed a greater penetration of tetramethylrhodamine-labeled BSA into the tumor parenchyma compared with the vessels in p-LKO tumors (Supplemental Figure 4, I and K). Together, the results show that the vasculature of SHP2-silenced tumors recapitulated features of “normalized” tumor vessels (26).

Importantly, we detected conspicuous p-Erk1/2^T202/Y205^ (Figure 4, C and D) and p-CDK2^T160^ (Figure 4, E and F) immunostaining in the viable islands of SHP2-silenced B16F10 tumors that was more intense than in the control tumors (Figure 4, D and F). Also, SHP2 immunostaining showed that the viable “islands” within SHP2-silenced B16F10 tumors continued to be SHP2 depleted compared with the control tumors (Figure 4, G and H), consistent with the effective SHP2 depletion documented by immunoblotting of these tumors (Figure 1D).

Additionally, Hypoxyprobe detected well-defined areas of hypoxia limiting the periphery of the Ki67^+^ tumor islands (Figure 4I). Thus, tumors derived from SHP2-silenced B16F10 cells displayed a subverted tissue architecture with clustering of highly proliferative tumor cells into islands around quiescent blood vessels with reduced branching and increased caliber, surrounded by areas of hypoxia and the demise of tumor tissue separating the viable islands. These phenotypic characteristics of SHP2-silenced B16F10 tumors resembled the SHP2-silenced B16F10 cells in 3D culture systems. Thus, a potential explanation for the formation of tumor islands in SHP2-silenced B16F10 tumors is that most tumor vessels are initially pruned, a phenomenon perhaps attributable to a localized deficiency of signals promoting endothelial cell survival (26), while other vessels undergo remodeling, providing a focal source of nutrients and oxygen and leading to clustering of proliferative cancer cells surrounding the remodeled vessels and to the death of cancer cells surrounding the dead/pruned vessels.

Therefore, we examined whether vessel remodeling per se drives the formation of tumor islands in SHP2-silenced B16F10 tumors. To induce tumor vessel remodeling, we targeted VEGF/VEGFR signaling with VEGF-Trap (27). Expectedly, low-dose VEGF-Trap (5 mg/kg) reduced B16F10 tumor growth, tumor weight, and tumor vascularization (Supplemental Figure 5, A–C). It also enhanced smooth muscle actin (SMA) and collagen IV (Col IV) coverage of CD31^+^ tumor vessels (Supplemental Figure 5, D–F), which are features linked to tumor vessel “normalization” (26). However, VEGF-Trap treatment did not induce the formation of B16F10 tumor islands surrounding vessels (Supplemental Figure 5G). These results, and the absence of reports linking vessel remodeling to clustering of tumor cells surrounding normalized vessels, suggest that the formation of tumor islands was not simply a consequence of vessel remodeling.

### SHP2 regulates angiogenesis-related proteins in B16F10 cells.

**Figure 5:**
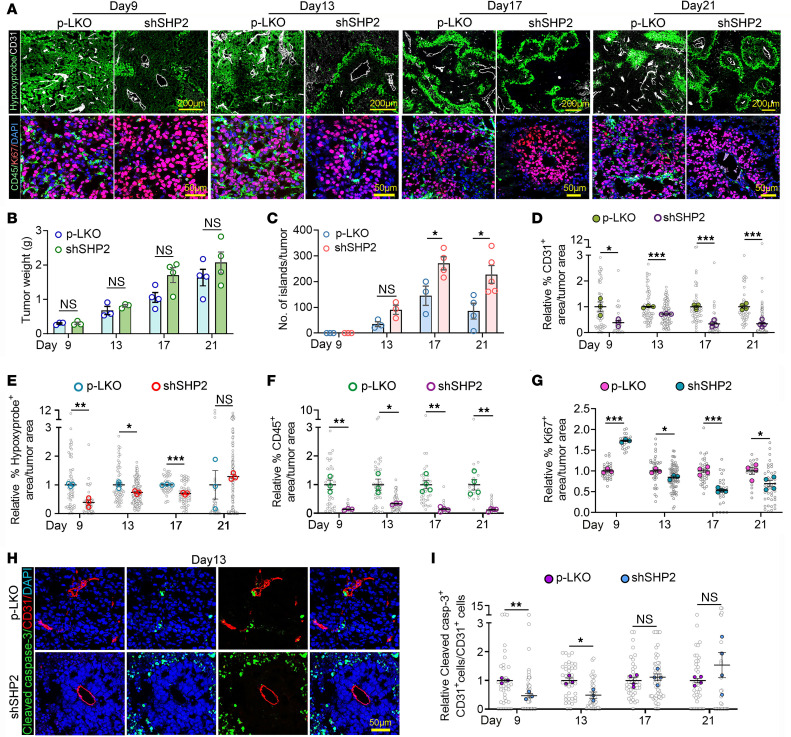
Kinetics analysis and characterization of tumor island formation. (**A**) Representative images of tumors harvested on days 9, 13, 17, and 21 following inoculation of p-LKO control and SHP2-silenced B16F10 cells. Top panels (scale bars: 200 μm) depict CD31^+^ vessels and distribution of hypoxia (Hypoxyprobe^+^); bottom panels (scale bars: 50 μm) show the distribution CD45^+^ inflammatory cells, Ki67^+^ proliferating cells, and cell nuclei (DAPI^+^). (**B**) Tumor weight of p-LKO and SHP2-silenced B16F10 tumors at the indicated time points; open circles represent individual tumors/mouse. *n* = 3 (days 9 and 13); *n* = 4 (days 17 and 21). (**C**) Quantification of tumor islands. A tumor island is defined as the assembly of live tumor cells around a blood vessel and surrounded by a zone of hypoxia. Quantification was performed by evaluating the entire tumor section through the maximum diameter; open circles represent individual tumors/mouse. *n* = 3 (days 9 and 13); *n* = 4 (days 17 and 21). *P < 0.05, by 2-tailed Student’s *t* test. (**D**–**G**) Quantification of CD31^+^ endothelial cells (**D**), hypoxic cells (Hypoxyprobe^+^) (**E**), CD45^+^ inflammatory cells (**F**), and Ki67^+^ proliferating cells (**G**) at the indicated time points in control tumors (*n* = 3, days 9 and 13; *n* = 4, days 17 and 21) and SHP2-silenced tumors (*n* = 3, days 9 and 13; *n* = 4, days 17 and 21). Gray circles: percentage of relative positive area/unit of tumor area; colored dots/circles: relative mean percentage of positive area/tumor. *P < 0.05, **P < 0.01, and ***P < 0.001, by 2-tailed Student’s *t* test. (**H** and **I**) Representative images (scale bar: 50 μm) from immunostaining of p-LKO (*n* = 3) and SHP2 silenced (*n* = 3) B16F10 tumor tissues harvested on day 13 for cleaved caspase 3 (cell death), CD31 endothelial cells, and nuclei (DAPI) (**H**) and quantification of cleaved caspase 3^+^CD31^+^ endothelial cells at the indicated time points following tumor cell inoculation (**I**). Gray circles: relative cleaved caspase 3^+^CD31^+^ cells/CD31^+^ cells analyzed; colored dots: tumor mean (control, *n* = 3; SHP2-silenced, *n* = 3). *P < 0.05 and **P < 0.01, by 2-tailed Student’s *t* test (**I**) The results in panels **B**–**G** and **I** are presented as means± SEM.

To gain mechanistic insights into tumor island formation, we first analyzed the kinetics of the process during tumor development (Figure 5, A and B). On day 9 after injection of B16F10 cells, virtually no tumor islands were observed (Figure 5, A and C), but SHP2-silenced tumors already differed from p-LKO tumors in that they showed a more structured and less abundant vasculature with reduced branching (Figure 5, A and D). Tumor hypoxia (Figure 5, A and E) and CD45^+^ cell infiltrates (Figure 5, A and F) were also reduced in SHP2-silenced tumors compared with p-LKO tumors on day 9, but the proportion of Ki67^+^ tumor cells was increased in SHP2-silenced tumors compared with that in p-LKO tumors (Figure 5, A and G). Beginning on day 13, some tumor islands became apparent (Figure 5, A and C), and their number increased up to day 17 before stabilizing by day 21, and this was associated with a progressive structural definition toward the fully developed tumor islands extending to large portions of the tumor (Figure 5A). In addition to displaying proliferative Ki67^+^ tumor cells surrounding a central tumor vessel, the tumor islands in SHP2-silenced tumors were surrounded by a well-defined hypoxic zone and cleaved caspase 3^+^ dead cells (Figure 5, A and H). Analysis of endothelial cell death within the CD31^+^ tumor vessels showed that, initially (day 9 and day 13), cell death was greater in the tumor vessels of p-LKO tumors than in SHP2-silenced tumors but was similar at the later time points (Figure 5I).

**Figure 6:**
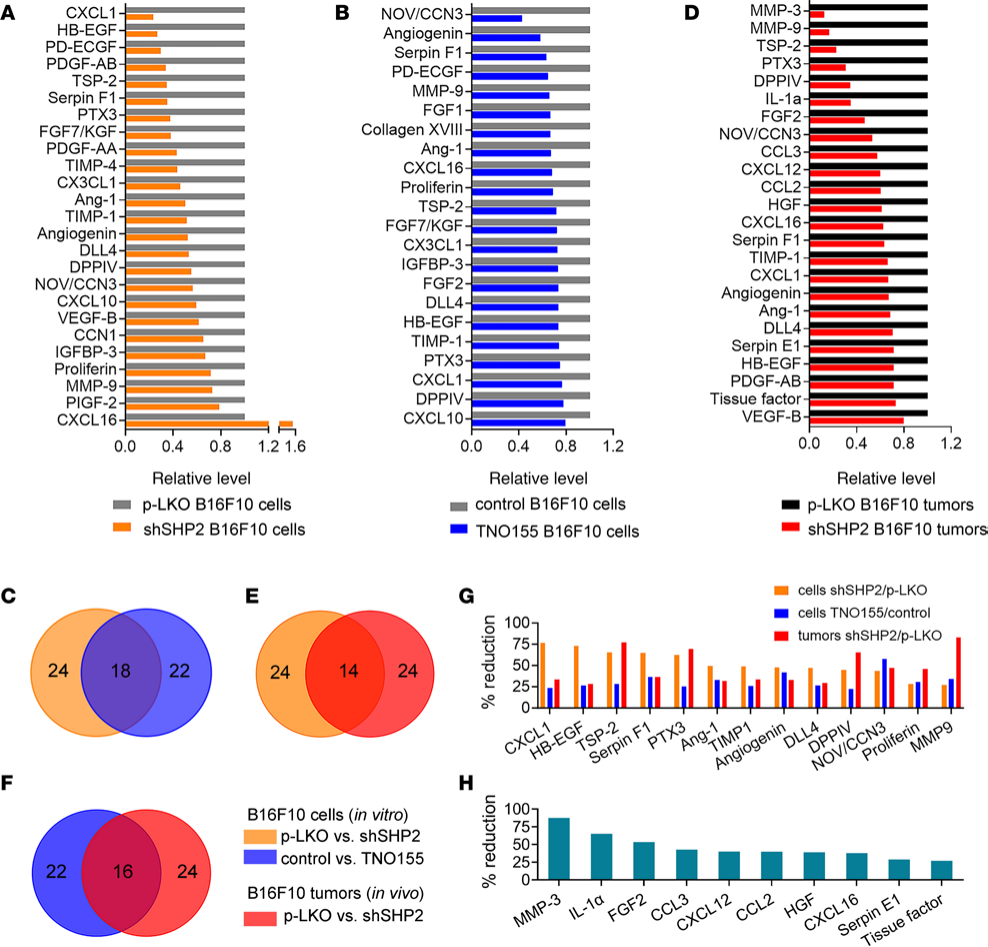
Effects of SHP2 depletion on angiogenesis regulatory proteins. (**A**) List of proteins that differed by more than 20% in relative pixel intensity between SHP2-silenced and p-LKO B16F10 cells. Cell lysates (900 mg) were applied to the array. (**B**) List of proteins with reduced pixel intensity of greater than 20% in TNO155-treated relative to control B16F10 cells. (**C**) Venn diagram of protein distribution. Some (*n* = 18) proteins reduced by more than 20% by SHP2 silencing in B16F10 cells were also reduced by more than 20% by TNO155 treatment of B16F10 cells. (**D**) List of proteins reduced by more than 20% in SHP2-silenced B16F10 tumors compared with p-LKO tumors removed from mice 21 days after inoculation. Lysates (900 mg) from pools of 4 control and 4 SHP2-silenced tumors were applied to the array. (**E** and **F**) Venn diagrams of protein distribution. Some proteins reduced by more than 20% in SHP2-silenced B16F10 tumors compared with control were also reduced by more than 20% by SHP2 silencing (**E**) or TNO155 treatment (**F**) of B16F10 cells from culture. (**G**) Proteins reduced by more than 20% compared with controls in SHP2-silenced B16F10 cells, TNO155-treated B16F10 cells*,* and SHP2-silenced B16F10 tumors. (**H**) Proteins were selectively reduced by more than 20% in SHP2-silenced B16F10 tumors compared with the control, but not in B16F10 cells from culture (SHP2-silenced compared with control and TNO-treated compared with control).

Since these kinetic experiments revealed that the distinctive vascular changes in SHP2-silenced B16F10 tumors preceded island formation, we broadly characterized the effects of SHP2 silencing on the output of vascular regulators in B16F10 cells. First, we profiled angiogenesis-related proteins in SHP2-silenced (shmL2) B16F10 cells from culture (Proteome Profiler Array for mouse angiogenesis, ARY015, R&D Systems). We found that the levels of 24 of 35 proteins detected (53 proteins total) were reduced by more than 20% in SHP2-silenced cells compared with the control (Figure 6A and Supplemental Figure 6A), including the proangiogenic factors HB-EGF (28), PD-ECGF/TYMP (29), PDGF-AA (30), PDGF-AB (30), angiogenin (31), Nov/IgfBP-9/CCN3 (32), and VEGF-B (33); the antiangiogenic proteins TSP2 (34), serpin F1 (35), the Notch ligand Dll4 (36), and CXCL10 (37); and other proteins with context-dependent vascular functions, including FGF-7/KGF (38), angiopoietin 1 (39), and TIMP1 (40). Only the multifunctional chemokine CXCL16 (41, 42) was increased in SHP2-silenced B16F10 cells compared with the control. Overall, these results show that SHP2 silencing reduced the levels of several vascular regulators expressed in B16F10 cells, potentially driving reduced tumor vascularity, vessel remodeling, formation of tumor islands, and other changes in SHP2-silenced tumors. The complexity of vascular factors regulated by SHP2 in B16F10 cells may explain why inhibition of VEGF/VEGFR2 only did not lead to the formation of tumor islands (Supplemental Figure 5G).

We examined potential mechanisms by which SHP2 silencing could reduce the output of vascular regulators in B16F10 cells (Figure 6A and Supplemental Figure 6A). Since p-AKT^S473^ was active in SHP2-silenced B16F10 cells from culture and tumors but not in p-LKO B16F10 cells and tumors (Figure 2A and Supplemental Figure 2A), and PI3K/AKT signaling can be either pro- or antiangiogenic by modulating the expression of angiogenic factors (43, 44), we sought to determine if AKT signaling contributes to reducing the expression of the identified vascular regulators. We found that the ATP competitive inhibitor AZD2014, which impairs AKT phosphorylation at S473 (45), reduced p-AKT^S473^ in SHP2-silenced B16F10 (Supplemental Figure 6B) and increased (>20%) the levels of 10 of 24 angiogenic regulators that were reduced in SHP2-silenced B16F10 cells compared with p-LKO control (Supplemental Figure 6C), including TSP2, CX3CL1, DLL4, and PPPIV proteins, which are known to be inhibited by the PI3K/AKT pathway in endothelial and other cell types (46–48). To a lesser degree (10%–20%), AZD2014 also increased the levels of an additional 7 factors (CXCL1, HB-EGF, PD-ECGF, PDGF-AB, PDGF-AA, angiopoietin 1, and VEGF-B) in SHP2-silenced B16F10 cells, but minimally changed the levels of the remaining 7 factors (FGF7/KGF, CXCL10, CCN1, IGFPB3, proliferin, MMP-9, and PlGF-2) (Supplemental Figure 6C). These results indicate a mechanistic role of p-AKT in reducing the levels of certain angiogenic regulators in SHP2-silenced B16F10 tumors.

In additional experiments, we analyzed the effects of the irreversible SHP2 inhibitor TNO155 (10 mM), which minimally alters B16F10 cell proliferation in vitro (Supplemental Figure 6D), on the output of angiogenic regulators from SHP2-silenced B16F10 cells. TNO155 reduced (>20%) the production of most of the proangiogenic (11 of 16), antiangiogenic (4 of 4), and context-dependent vascular factors (3 of 4) reduced by SHP2 silencing in B16F10 cells (Figure 6, B and C, and Supplemental Figure 6, E and F). Unlike the silencing of SHP2, TNO155 additionally reduced the levels of the proangiogenic factors FGF1 and FGF2 (49) in B16F10 cells.

We extended this analysis to another tumor cell line (the human acute myeloid leukemia cell line MOLM13) that is growth resistant with the SHP2 inhibitor SHP099 after KO of the *INPPL1* or *MAP4K5* genes (7) (GSE218491, GSE21223). Consistent with the results from SHP2 depletion or TNO155 treatment of B16F10 cells, we found that with SHP099 treatment, growth-resistant MOLM13 cells had downregulated (>20%) expression of several genes coding for the same proangiogenic proteins that were reduced in SHP2-silenced B16F10 cells (Supplemental Figure 6G). Expression of the principal proangiogenic factor, *VEGFA*, was also reduced by SHP099 in growth-resistant MOLM13 cells (Supplemental Figure 6G).

Thus, depletion or inactivation of SHP2 reduced the output of various proangiogenic factors in mouse and human tumor cells that were growth resistant to SHP2 silencing or inhibition.

### Host-tumor interactions regulate cytokine, chemokine, and growth factor production.

Next, we profiled angiogenic regulators in SHP2-silenced and control B16F10 tumors removed from mice when tumor vascularization was reduced compared with the control, and observed a distinctive architecture of tumor islands in the SHP2-silenced tumors (Figure 4 and Supplemental Figure 3, A and B). We again found that the expression levels of several proangiogenic (*n* = 19), antiangiogenic (*n* = 3), and context-dependent (*n* = 2) vascular factors were reduced (>20%) in SHP2-silenced (shmL2) tumor lysates compared with controls (Figure 6D and Supplemental Figure 6H), a sizable proportion of which were the same factors reduced in SHP2-depleted (Figure 6E and Supplemental Figure 6I) and TNO155-treated (Figure 6F) B16F10 cells from in vitro culture. Overall, 13 angiogenic regulators were reduced in all 3 groups (Figure 6G).

Tumors produced by inoculation of B16F10 cells differed from B16F10 cells derived from culture, as there were 10 additional factors detected at lower levels in the SHP2-silenced B16F10 tumors compared with p-LKO controls (Figure 6D). These factors included the proinflammatory cytokine IL-1α, an alarmin responsive to various sterile signals (damage-associated molecular patterns [DAMPs]) (50, 51), the effector chemokines CCL2 (also known as MCP1) (52, 53), CCL3 (also known as MIP1a) (54), CXCL12 (55), and CXCL16 (56), the proangiogenic hepatocyte growth factors HGF and FGF2 (57, 58), the metalloproteinase MMP3 (also known as stromesilyn 1) (59), and the regulators of blood coagulation Serpin E1 (60) and tissue factor (61) (Figure 6, D and H).

**Figure 7:**
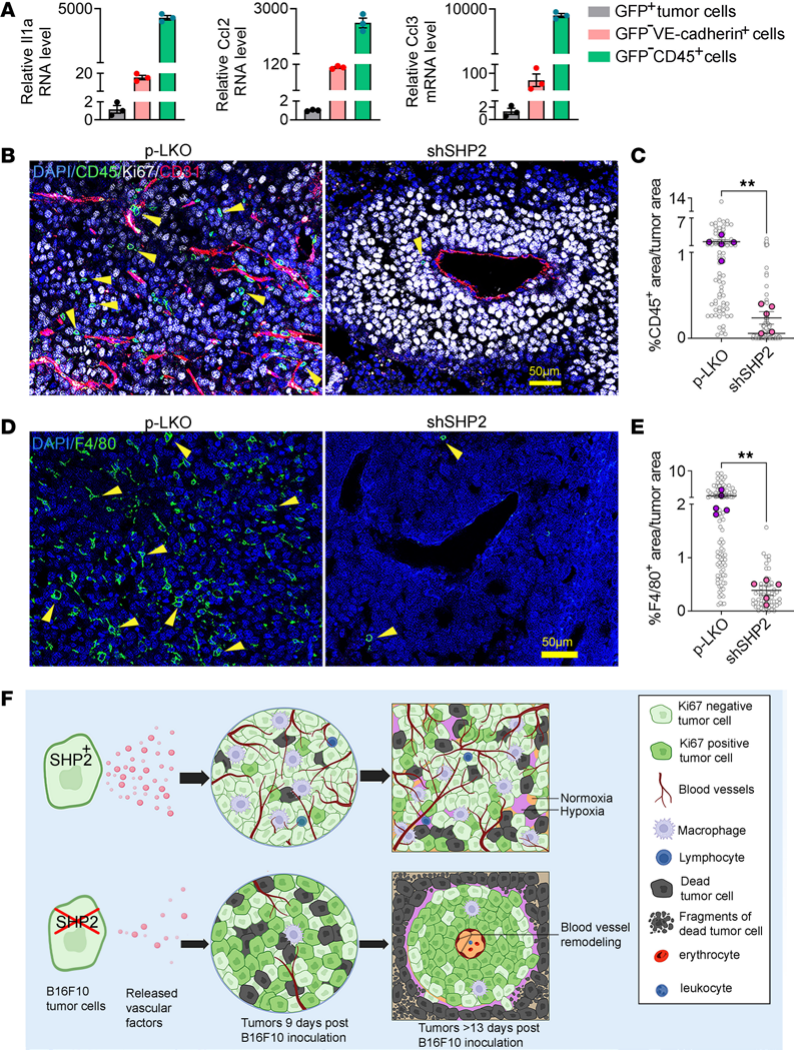
SHP2 regulation of angiogenesis-related genes in the B16F10 tumor microenvironment. (**A**) Expression levels of *Il1a*, *ccl2*, and *ccl3* in sorted populations of EGFP^+^ B16F10 cells, EGFP^–^VE-cadherin^+^ endothelial cells, and EGFP^–^CD45^+^ hematopoietic cells as measured by qPCR. The results are expressed as the relative mean ± SEM of *Gapdh* mRNA levels (*n* = 3 experiments). (**B** and **C**) Representative images (scale bar: 50 μm) (**B**) showing CD45^+^ cell infiltrates (green, indicated by arrowheads) in control and shSHP2-silenced B16F10 tumors and quantification (**C**). Gray circles: percentage of CD45^+^ area/unit of tumor area; colored dots: mean/tumor (*n* = 5/group). **P < 0.01, by 2-tailed Student’s *t* test. (**D** and **E**) Representative images (scale bar: 50 μm) (**D**) showing F4/80 (green) macrophage infiltration in control and shSHP2-silenced B16F10 tumors (indicated by arrowheads) and quantification (**E**). Gray circles: percentage of F4/80^+^ area/unit of tumor area; colored dots: mean/tumor (*n* = 5/group). **P < 0.01, by 2-tailed Student’s *t* test. (**F**) Proposed model of downstream effects from SHP2 silencing in B16F10 melanoma cells leading to the formation of vascular tumor islands and surrounding tumor cell death. Results in panels **A**, **C** and **E** are presented as means± SEM.

The genes coding for the unique factors detected in B16F10 tumors, but not in B16F10 cells from culture, are essentially not expressed in B16F10 cells from culture or are expressed at much lower levels than in B16F10 tumors (GSE212112), suggesting derivation from the tumor microenvironment. To identify the cell source of these factors, we sorted B16F10 tumor cells, VE-cadherin^+^ endothelial cells, and CD45^+^ hematopoietic cells from a B16F10 tumor produced by EGFP^+^ B16F10 cells (Supplemental Figure 7A). By quantitative PCR (qPCR), we confirmed that *Il1a*, *Ccl2*, and *Ccl3* mRNAs were virtually undetected in the tumor cells but were expressed predominantly in the CD45^+^ hematopoietic cells and, to a lesser degree, in the VE-cadherin^+^GFP^–^ endothelial cells (Figure 7A). By single-cell RNA-Seq, we detected *Cxcl12*, *Mmp3*, *Hgf*, *Serpine1*, *F3*, *Fgf2*, and *Cxcl16* mRNAs predominantly in B16F10 tumor–associated stromal cells expressing the genes *Col1a1*, *Col1a2*, and *Sparc*; to some degree, the *Cxcl12*, *Hgf*, *Serpine1*, and *Cxcl16* genes were also expressed in CD31^+^ endothelial cells; additionally, *Hgf* and *Cxcl16* genes were also expressed in CD45^+^ inflammatory cells (62).

Since CCL2, CCL3, and CXCL12 chemoattract monocytes, macrophages, and other cell types (63, 64), we examined the inflammatory cell populations in B16F10 tumors. Consistent with the reduction of IL-1α, CCL2, and CCL3 in SHP2-silenced tumors (Figure 6H and Supplemental Figure 7, B and C), IHC revealed that tumor-infiltrating CD45^+^ hematopoietic cells and F4/80^+^ macrophages were significantly reduced in SHP2-silenced B16F10 tumors compared with controls (Figure 7, B–E). CD3^+^ T and CD19^+^ B lymphocytes were rarely detected in control or SHP2-silenced B16F10 tumors (Supplemental Figure 7D).

Overall, our results outline processes by which SHP2-silenced B16F10 cells gave rise to tumors with distinctive tumor islands (Figure 7F). SHP2-silenced tumor cells produced reduced levels of a heterogeneous set of vascular regulators, cytokines, and chemokines compared with the control, and tumors derived from these SHP2-silenced cells were less vascularized, the vessels were remodeled, and proinflammatory cells were fewer. Also, the SHP2-silenced tumor cells acquire a propensity to cluster and to be more dependent on oxygen/nutrients compared with controls, changes that together promote tumor cell assembly around remodeled vessels where they thrive, whereas cells at a distance from the remodeled vessels become hypoxic and die.

Key events associated with SHP2 silencing in B16F10 tumors were recapitulated in SHP2-silenced MC38 colon cancers (Figure 1, H–N) (11). SHP2-depleted MC38 tumors were significantly less vascularized than those derived from p-LKO control tumors (Figure 1, L and M) and structurally differed from controls in showing clusters of proliferating tumor cells surrounding normal-appearing tumor vessels separated by nonvascularized tumor composed on nonproliferating tumor cells (Supplemental Figure 8, A and B). The content of several angiogenic and proinflammatory factors, including VEGFA and FGF2, was reduced in other SHP2-silenced tumor cells from culture and the tumors they generated (Supplemental Figure 8, C and D). Additionally, IL-1α, CCL3, CXCL12, and HGF levels were reduced in SHP2-silenced tumors compared with controls (Supplemental Figure 8D), which was associated with a reduction of CD45^+^, F4/80^+^, and CD3^+^ cell infiltrates (Supplemental Figure 8, E–H). Thus, SHP2 silencing in tumor cells that grow despite silencing SHP2 can regulate the tumor vasculature and other components of the tumor microenvironment.

### Relevance of the experimental mouse melanoma model to human melanoma.

**Figure 8:**
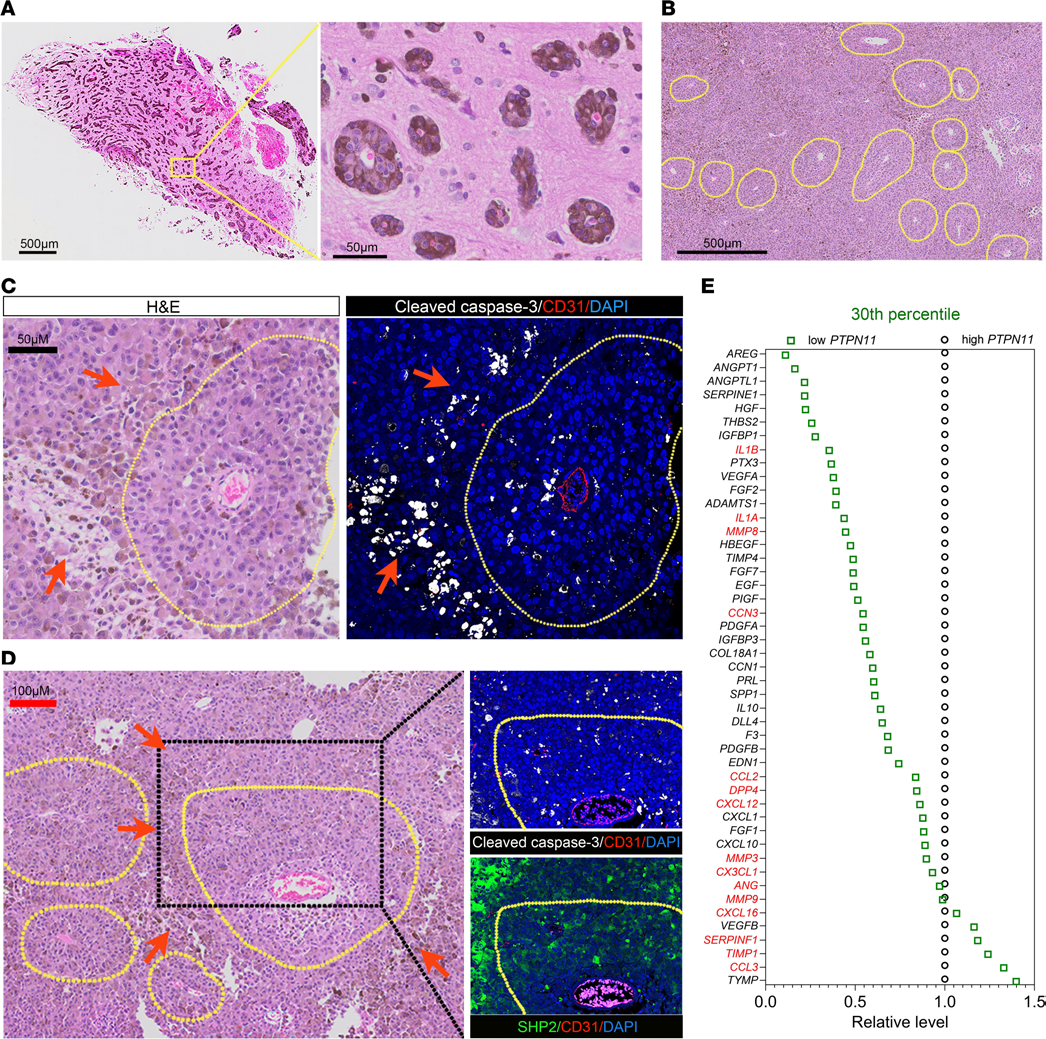
Relevance of SHP2-silenced B16F10 mouse tumor model to human melanoma. (**A**) H&E-stained histologic images of human angiotropic melanoma metastasized to the brain. The rectangular area outlined on the left is magnified on the right. Scale bars: 500 μm (left) and 50 μm (right). (**B**) H&E-stained histologic low-magnification image of a primary cutaneous angiotropic melanoma. Scale bar: 500 μm. (**C**) Confocal immunofluorescence images of a primary cutaneous angiotropic melanoma (H&E-stained, left) and immunostaining (right) for CD31 (endothelial cells) and cleaved caspase 3 (cell death). DAPI identified cell nuclei (right). The yellow dotted line outlines a tumor area surrounding a blood vessel and peripherally limited by less viable tumor. Red arrows in panels C and D point to areas of tumor cell death. Scale bar: 50 μm. (**D**) H&E-stained confocal images showing a typical primary cutaneous angiotropic melanoma (left). The area outlined by the black dotted line is shown on the right after immunostaining for CD31 and cleaved caspase-3 (top) and SHP2 and CD31 (bottom); DAPI depicts cell nuclei. Scale bar: 100 μm. (**E**) Relative expression levels of the indicated genes in human cutaneous melanoma (TCGA-SKCM) expressing either high (30%) or low (30%) *PTPN11* levels (*n* = 142/group). Each dot represents the relative mean expression level; expression of the genes in red was not statistically different in the 2 groups (2-tailed Student’s *t* test).

Previous studies identified a rare type of human melanoma, “angiotropic melanoma,” which is characterized histologically by melanoma cell cuffing, but not invasion, of the external wall of intact vessels, thereby forming angio-tumoral complexes (65, 66). Angiotropic melanoma from brain metastasis (Figure 8A) and primary cutaneous melanoma (Figure 8B) resembled mouse B16F10 tumors. A typical island from cutaneous angiotropic melanoma was limited by areas where the tumor cells were smaller in size, displayed nuclear fragmentation/karyorrhexis (indicated by arrows in Figure 8C), and expressed the death marker cleaved caspase 3^+^, whereas mostly viable tumor cells surrounded a well-defined CD31^+^ blood vessel (Figure 8C). Another typical tumor island, demarcated by a rarefied tumor structure (indicated by arrows in Figure 8D) in which cells were positive for cleaved caspase 3, was composed of mostly viable (cleaved caspase 3^–^) tumor cells (Figure 8D), which expressed lower levels of SHP2 than did viable cells at the periphery of the island (Figure 8D). These results show that human angiotropic melanoma resembled B16F10 mouse melanomas with a similar island structure limited by less viable tumor cells and that within the islands, viable tumor cells clustered around a perfused blood vessel and expressed low levels of SHP2.

Since transcriptomic datasets from human angiotropic melanomas are not currently available, we mined public transcriptome datasets of human skin melanoma (The Cancer Genome Atlas–skin cutaneous melanoma [TCGA-SKCM], where “angiotropic melanoma” is not annotated) to examine relationships between expression levels of *PTPN11* (codes for the SHP2 protein) and expression levels of vascular regulators analyzed in mouse B16F10 tumors. To approximate SHP2-silenced B16F10 tumors, we selected human melanomas expressing the 10%–50% highest and 10%–50% lowest *PTPN11* levels. In all groups examined, *PTPN11* expression directly correlated with expression levels of many of the same vascular factors regulated by SHP2 silencing in B16F10 cells and tumors (Figure 8E and Supplemental Figure 9, A–D).

Also, we found that *PTPN11* expression was significantly higher in metastatic compared with primary melanoma tissues (Supplemental Figure 9E), linking high *PTPN11* expression with disease progression. Furthermore, several vascular regulators reduced in SHP2-silenced mouse B16F10 cells and tumors were expressed at significantly higher levels in metastatic compared with primary human melanomas, including *ANGPT1*, *THSB2* (codes for thrombospondin 2), *PTX3*, *FGF7* (also known as *KGF*), and *VEGFA* (Supplemental Figure 9, F–V). These results are consistent with a contribution of angiogenic regulators to a proposed oncogenic role of SHP2 in human melanoma (67, 68).

### Antitumor efficacy of targeting MEK/ERK in SHP2-silenced B16F10 melanoma.

SHP2 depletion in B16F10 cells affected their growth as tumors in mice. A key feature of these tumors was the clustering of highly proliferative tumor cells in vascular islands, where the MAPK/ERK/CDK2 signaling pathway was active, more active than in control SHP2^+^ tumors (Figure 2C, Figure 3, A–C, Figure 4, and Supplemental Figure 2B).

**Figure 9:**
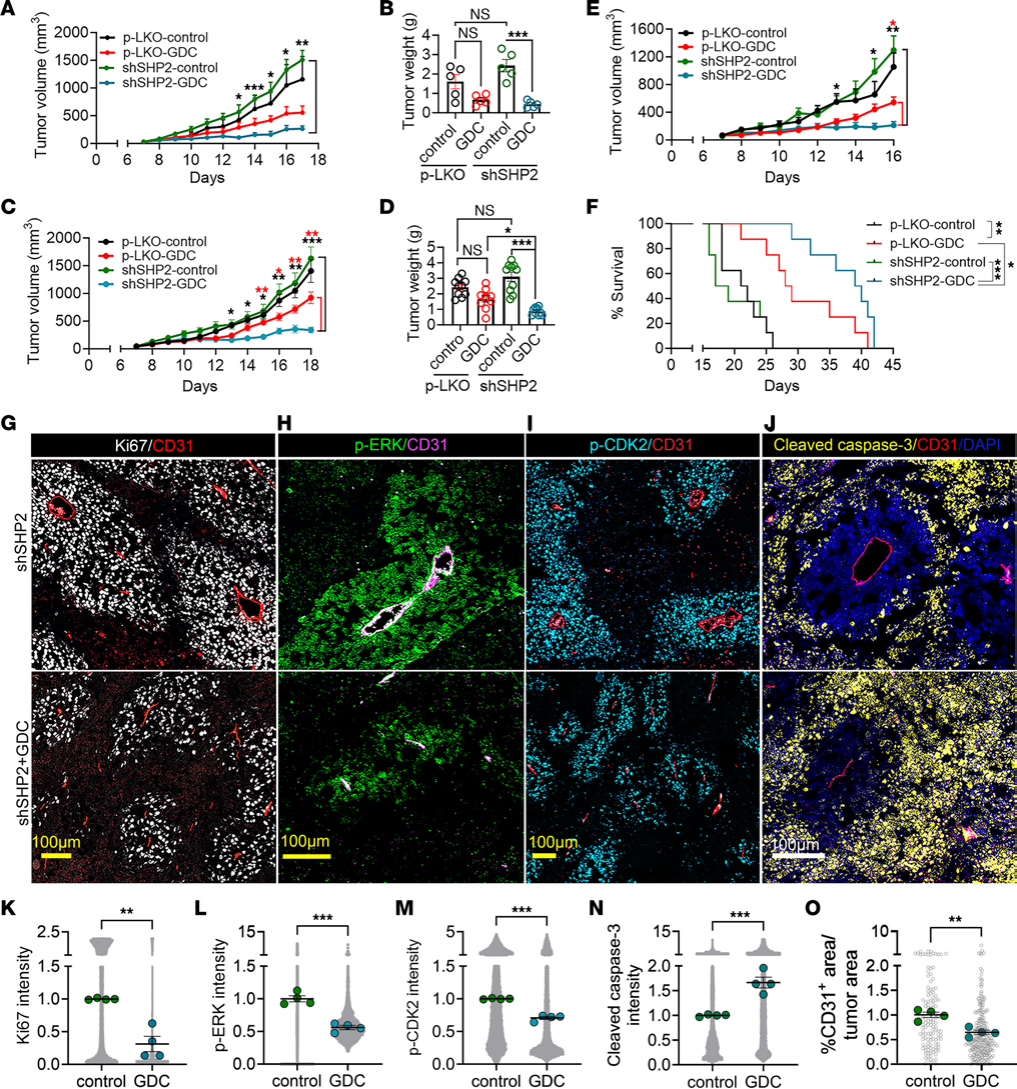
Contribution of SHP2 depletion to the antitumor activity of the MEK inhibitor GDC-0623. (**A**–**D**) Tumor-bearing mice (*n* = 5/group in **A** and **B**; *n* = 10/group in **C** and **D**) from s.c. inoculation of SHP2-silenced or control (p-LKO) B16F10 cells were treated with 30 mg/kg daily oral GDC-0623 (GDC) or buffer only. The experiment was terminated when the maximal tumor diameter reached 20 mm in any mouse. Tumor volumes (**A** and **C**) for shSHP2-GDC versus shSHP2-control (black asterisks) and shSHP2-GDC versus p-LKO–GDC (red asterisks). Tumor weights (**B** and **D**) at harvest. (**E** and **F**) Tumor-bearing mice (*n* = 8/group) were s.c. inoculated with SHP2-silenced or control B16F10 cells and treated with 30 mg/kg daily oral GDC-0623 or buffer only. Each mouse was euthanized when the endpoint (20 mm maximum diameter or humane endpoint) was reached. (**E**) Tumor size measurements from treatment initiation to the day when the first mouse reached the endpoint; shSHP2 control versus shSHP2-GDC (black asterisks); p-LKO–GDC versus shSHP2-GDC (red asterisk). (**F**) Proportion of mice surviving as a function of time from the beginning of treatment (Kaplan-Meier curves). *P < 0.05, **P < 0.01, and ***P < 0.001, by 1-way ANOVA (**B** and **D**) and 2-way ANOVA (**A**, **C**, and **E**) for multiple comparisons. (**G**–**O**) Tumors from inoculation of SHP2-silenced B16F10 cells were treated with GDC-0623 or buffer only. Tumor sections were visualized by confocal imaging after immunostaining (representative images in **G**: Ki67/CD31; **H**: p-ERK^T202Y204^/DAPI; **I**: p-CDK2^T160^/DAPI; **J**: cleaved caspase 3/CD31/DAPI). Scale bars: 100 μm. The relative fluorescence intensity of specific markers/DAPI^+^ cells was quantified (**K**: Ki67; **L**: p-ERK^T202Y204^; **M**: p-CDK2^T160^; **N**: cleaved caspase 3; **O**: CD31; gray circles, appearing as gray zones when numerous, reflect the positive area/unit of tumor area; green dots indicate the mean/tumor (*n* = 4/group). **P < 0.05 and ***P < 0.001, by 2-tailed Student’s *t* test. Horizontal lines indicate the mean. Results in panels **A**–**E** and panels **K**–**O** are presented as means± SEM.

Exploiting these changes, we tested the antitumor efficacy of the MEK inhibitor GDC-0623 (69), which specifically inhibits MEK/ERK signaling. Therefore, tumors that developed in mice inoculated with SHP2-silenced or control (p-LKO) B16F10 melanoma cells were treated with either GDC-0623 or buffer only (Figure 9, A–F). GDC-0623 (30 mg/kg, daily, orally) significantly reduced the growth of SHP2-silenced B16F10 tumors at different time points after inoculation of the SHP2-silenced tumor cells and, to a smaller, insignificant degree, the growth of control (p-LKO) tumors (2-way ANOVA for multiple comparisons; Figure 9, A, C, and E). Similarly, GDC-0623 significantly reduced the weight of SHP2-silenced tumors, but not control tumors, at the endpoint (Figure 9, B and D). In the experiments shown in Figure 9, A–D, all mice were euthanized when a tumor in any mouse in any group reached the maximum diameter of 20 mm in any direction, on days 17 and 18. Although GDC-0623 insignificantly inhibited the growth of B16F10 melanoma tumors as previously observed (70), our results suggested that GDC-0623 could show effectiveness against B16F10 melanoma after extended treatment. An additional experiment examined the effects of GDC-0623 on mouse survival. Per protocol, euthanasia was mandated for individual mice when the tumor size reached the maximum caliper measurement (20 mm in any direction) or at humane endpoints. The size of tumors on day 16 (Figure 9E), when a tumor in a single mouse reached the maximum diameter, closely recapitulated the results in Figure 9, A and C. Continued treatment until the euthanasia endpoint showed that GDC-0623 was effective at extending the survival of mice bearing p-LKO tumors (*P* < 0.01, log-rank test) but more effective at prolonging survival of mice with SHP2-silenced (*P* < 0.001) B16F10 tumors (*P* < 0.05). Body weights of GDC-0623–treated or diluent-only-treated mice were similar (Supplemental Figure 10A), suggesting good drug tolerance. The greater effect of GDC-0623 on the growth of SHP2-silenced tumors and survival of mice bearing SHP2-silenced tumors compared with controls contrasts with the results in vitro showing that GDC-0623 similarly reduced the proliferation of SHP2-silenced and control (p-LKO) B16F10 cells (Supplemental Figure 10B), suggesting the emergence of increased drug sensitivity in vivo*,* perhaps linked to increased MAPK/ERK signaling in SHP2-silenced tumors compared with controls.

Treatment with GDC-0623 did not subvert the island structure of SHP2-silenced B16F10 tumors, but the islands were generally smaller in size and more sparsely populated by cells (Figure 9G). The staining intensity of Ki67^+^, p-ERK^+^, and p-CDK2^+^ in the island-resident tumor cells was reduced after GDC-0623 treatment (Figure 9, G–I, and K–M), evidence that the drug had on-target effects and reduced tumor cell proliferation. Also, the cell death marker cleaved caspase 3, which typically identifies areas surrounding the islands in SHP2-silenced B16F10 tumors, was more broadly diffused in GDC-0623–treated, SHP2-silenced B16F10 tumors (Figure 9, J and N). In addition, compared with the controls, GDC-0623 reduced tumor vascularization in SHP2-silenced B16F10 tumors (Figure 9, I and O), and the vessels appeared to have a reduced lumen, but were perfused (Supplemental Figure 10C).

Control (p-LKO) B16F10 tumors also showed changes after treatment with GDC-0623 (Supplemental Figure 10, D–K), including a significant reduction of Ki67, p-ERK, and p-CDK2 (Supplemental Figure 10, E, G, and I) and an increase in cleaved caspase 3 immunostaining intensity (Supplemental Figure 10K). In addition, GDC-0623 treatment resulted in an insignificant reduction of tumor vascularization and F4/80^+^ cell infiltration in p-LKO B16F10 tumors, but a significant reduction of tumor infiltration with CD45^+^ cells (Supplemental Figure 10, L–N). Furthermore, infiltration of CD45^+^ or F4/80^+^ cells, which is very low in SHP2-silenced B16F10 tumors compared with p-LKO controls (Figure 5, B–E), was virtually unchanged by GDC-0623 (Supplemental Figure 10, M and N). These results show that SHP2 depletion in B16F10 melanoma enhanced responsiveness to the MEK/ERK inhibitor GDC-0623, despite SHP2 depletion having no effect on B16F10 tumor growth.

## Discussion

In this study, we identify SHP2 as a complex regulator of the vascular phenotype and structural architecture of melanoma and colon carcinoma. Most cancer lines are known to produce proangiogenic factors and other vascular regulators that drive tumor angiogenesis critical to tumor growth. We found that depletion or functional inhibition of SHP2 in melanoma and colon carcinoma cells, themselves independent of SHP2 for proliferation in vitro, broadly reduced the output of factors that regulate tumor vascularization. Thus, tumors originating from SHP2-depleted B16F10 melanoma and MC38 colon carcinoma cells displayed a distinctive signature with reduced vascularization, a restrained proinflammatory phenotype, and a subverted architecture with remarkable clustering of tumor cells around selected blood vessels, forming islands of perivascular proliferative tumor tissue separated by dead/dying tumor tissue. Although vascular islands may reflect protected sanctuaries for tumor cells, we found that island-resident melanoma cells had enhanced ERK activity and increased responsiveness to MEK/ERK inhibition. This increased responsiveness to MEK/ERK inhibition may have been facilitated by increased drug delivery through the remodeled tumor vasculature. Thus, we provide a molecular model that accounts for a SHP2-controlled biochemical, structural, and functional evolution of experimental melanoma and colon cancers and offers a therapeutic opportunity. It remains to be established, however, if the combined SHP2 and MEK targeting is broadly applicable to overcome resistance to SHP2 inhibition.

A key finding of this work is that sensitization to inhibition of the MEK/ERK cascade can be achieved in cancer cells through mechanisms that rely on SHP2-controlled production of vascular factors, increased cell clustering, and increased dependence on nutrients and oxygen for cell survival. Several lines of evidence showed that SHP2 silencing, rather than an off-target effect of silencing, was the mechanism for modulation of angiogenic factors in our experiments. First, the specific SHP2 inhibitor TNO155 and SHP2-targeted shRNAs similarly modulated angiogenic factor output by the melanoma and colon carcinoma cells in vitro. Moreover, another SHP2 inhibitor, SHP099, reduced the expression of angiogenic factors in the human acute myeloid leukemia MOLM13 cells otherwise resistant to SHP2 inhibition (7) and altered the expression chemokines and cytokines in Kras^G12D^–driven p48-Cre; trp53^lox/lox^ (KPC) pancreatic tumors prompting an influx of T cells (71).

SHP2 is required for full activation of the RAS/RAF/MEK/ERK signaling pathway, acting upstream of RAS. Increased activity of the ERK pathway is a major driver of adaptive resistance to SHP2 inhibition in cancer cells, and combining SHP2 inhibitors with ERK inhibitors may prevent the development of drug resistance to MEK inhibitors (7, 10, 72, 73). Unlike these previous studies, our goal was not to prevent SHP2 adaptive resistance to inhibition of MEK/ERK signaling but rather to exploit SHP2 regulation of parallel pathways (e.g., AKT, JAK/STAT) that control the production of vascular regulators, cell adhesion, and cell survival, prompting a change of the tumor architecture to segregate tumor cells into vascular islands.

It is evident from many studies that multiple tumor cell–derived factors contribute to sustain proangiogenic tumor microenvironments, and this could explain why targeting one or a few factors often leads to compensation by other factors (74) and does not produce broad structural changes in cancer tissues. By targeting SHP2, an upstream regulator of multiple signaling regulatory pathways (75), we induced tumor vessel remodeling, clustering highly proliferative tumor cells into vascular islands, and reduced tumor inflammation.

The drug razoxane (ICRF-159) was observed to normalize the chaotic vasculature of Lewis lung cancer (24), followed by other strategies, including inhibition of VEGFA/VEGFR2 (76), PDGFRb (77), angiopoietin 2 (78), or the regulator of G protein signaling 5 (Rgs5) (79), and slowing endothelial cell glucose metabolism (80). The goal was to improve blood perfusion for better delivery of antitumor drugs and immune cell trafficking to the tumor, but “normalizing” the tumor vasculature remains a challenge (75).

An important question is how to further develop the current experimental approach of selectively targeting SHP2 in tumor cells, in isolation of other cells. This is important because the SHP2 inhibitor SHP099 can directly block endothelial cell proliferation, inhibit tumor neovascularization, induce tumor vessel involution, and promote vascular leakage, outcomes that differ from the effects of silencing SHP2 in the tumor cells (11). Reduced doses of SHP2 inhibitors might produce effects that resemble SHP2 depletion in the tumor cells, consistent with the differences between conventional versus judicious doses of angiogenic inhibitors producing regression versus normalization of tumor vessels (81).

It will be interesting to determine whether MEK inhibitors synergize with SHP2 inhibitors in other tumor types and, if so, whether identical or other tissue-specific mechanisms are engaged. Regardless, the data presented here provide compelling support for the development of new therapeutic strategies for the combined targeting of SHP2 and MEK in cancer types that have acquired new sensitivities to oncogenic inhibitors once tumor cells have developed SHP2 growth independence.

## Methods

### Sex as a biological variable.

We conducted this study using female mice as recipients of the mouse B16F10 (82), MC38 (83), and CT26 (84) tumor cell lines because these cells grow more slowly into tumors in female mice than in male mice. Our goal was to have a long treatment window from tumor appearance to euthanasia and to aim for experimental accuracy more easily achievable in the more docile female compared with male mice. Key findings are expected to be relevant in both sexes, as documented in previous experiments (11).

### Animal experiments.

We used female C57BL/6J mice (The Jackson Laboratory, mL000664) as recipients of the syngeneic tumor lines B16F10 (ATCC CRL-6475) and colon adenocarcinoma MC38 (a gift of James W. Hodge, Center for Cancer Research, NCI, Bethesda, Maryland, USA) and female BALB/cJ mice (Charles River Laboratories, code 028) as recipients of the murine colon carcinoma cell line CT26 (ATCC CRL-2638).

Female 8- to 10-week-old C57BL/6J mice were inoculated s.c. with B16F10 (0.02 × 10^6^ to 1 × 10^6^ cells) or MC38 (1 × 10^6^) cells, and 6- to 8-week-old female BALB/cJ mice were inoculated with CT26 (1 × 10^6^) cells in the abdomen. Tumor volume was estimated using the following formula: *V* = *D*(*d^2^*)/2 (*D* being the longest and *d* the shortest perpendicular dimensions). C57BL/6J or BALB/cJ mice bearing palpable s.c. tumors were separated into treatment groups according to tumor sizes. Mice were given a daily oral (via gavage) dose of 0.1 mL formulation buffer alone (0.5% Tween 80 [MilliporeSigma, no. P6224] and 0.5% Methyl Cellulose [MilliporeSigma, no. M0430] in distilled water) or a daily oral dose of GDC-0623 (MedChemExpress, no. HY-15610; 30 mg/kg) in 0.1 mL formulation buffer, or were twice weekly s.c. inoculated with 5 mg/kg VEGF-Trap (ZALTRAP, ziv-aflibercept, Sanofi-Aventis) in PBS or 0.1 mL PBS alone. Tumor size was measured daily, and body weight was measured prior to tumor cell inoculation and before the mice were euthanized. The following experimental endpoints were used: (a) All mice were euthanized when tumors (B16F10 or MC38 tumors) in any mouse from any experimental group reached or approached a size of 20 mm in any direction or developed a humane endpoint for euthanasia. (b) Mice were euthanized individually when the tumor (B16F10 tumors) reached or approached a size of 20 mm in any direction (the remaining mice were continued on treatment). (c) Mice in the different groups were euthanized at the predetermined post-inoculation time points of 9, 13, 17, or 21 days. (d) All mice were euthanized at a single, predetermined time point or tumor size (CT26 tumors) following inoculation. To measure blood perfusion, 100 μL PBS solution containing FITC-dextran (2,000,000 MW, 50 mg/mL, Thermo Fisher Scientific, no. D7137) and poly-l-lysine (300 kDa, 10 mg/mL, MilliporeSigma, no. P-1524) was injected retro-orbitally into mice. After 5–10 minutes, the mice were euthanized and tissues processed. To assess vessel permeability, tetramethylrhodamine-conjugated BSA (5 mg/mL, Thermo Fisher Scientific, no. A23016) was injected retro-orbitally into mice. After 1 hour, the mice were euthanized and tissues processed. To assess tissue hypoxia, 1.5 mg hypoxyprobe-1 (Hypoxyprobe, HP3-100) was administered orally to mice. After 60 minutes, the mice were euthanized, and tumor tissues were collected and processed for analysis.

### Cells, cell culture, and materials.

The murine melanoma B16F10 (American Type Culture Collection [ATCC], CRL-6475), colon adenocarcinoma MC38 (a gift of James W. Hodge, Center for Cancer Research, NCI, NIH), and human embryonic kidney 293T (ATCC, CRL-3216) cell lines were cultured in DMEM with 10% FCS. The murine colon carcinoma CT26 cell line (ATCC, CRL-2638) was cultured in RPMI-1640 medium with 10% FCS. TNO155 (Investigational Drugs Repository, NCI Division of Cancer Treatment and Diagnosis [DCTD] and MedChemExpress, mLHY-136173), GDC0623 (MedChemExpress, mLHY-15610), or AZD2014 (MedChemExpress, mLHY-HY-15247) inhibitors were solubilized in DMSO at 10 μM and then aliquoted and stored at –80°C. Further dilutions were done in PBS before addition to culture at varying concentrations (0.1–10 μM).

### Gene silencing and expression.

Lentiviral shRNA particles were generated for mouse SHP2 silencing (MISSION shRNA; MilliporeSigma, TRCN0000327987, TRCN0000029877, TRCN0000328059, TRCN0000029875, TRCN0000029878, and TRCN0000327984) and control p-LKO (MISSION shRNA Control Vectors; MilliporeSigma, SHC001) in 293T cells using the third-generation lentiviral packaging system. Infected cells were selected with puromycin (1 μg/mL for B16F10/CT26 cells, or 5 μg/mL for MC38 cells; Thermo Fisher Scientific, mLA11138) for 10 days. RNA purification, cDNA production, and real-time PCR are described in Supplemental Methods.

### Cell sorting, flow cytometry, and cell proliferation.

Sorting of GFP+ B16F10 tumor cells from single-cell suspensions and post-sorting analysis are described in Supplemental Methods. Cell proliferation was measured by ^3^H-thymidine incorporation as detailed in Supplemental Methods.

### In-vitro 3D suspension cultures and Matrigel-based island formation assays.

Control p-LKO and SHP2-silenced B16F10 cells were cultured (2 × 10^4^/well in DMEM with 10% FBS and 1 μg/mL puromycin) in low-attachment, 6-well plates (Corning, no. 3471) to induce growth in suspension (23). B16F10 cells (p-LKO and SHP2-silenced) were also incubated in a 3D Matrigel-based chamber assay system to detect in vitro island formation as detailed in Supplemental Methods.

### ELISAs, protein array assays, and Western blotting.

Protein kinase phosphorylation was measured in B16F10 tumor cell lysates using the Human Phospho-Kinase Array Kit (R&D Systems, mLARY003C). This phospho-kinase array, designed for human samples, has been successfully used for mouse samples (72). Angiogenesis-related proteins from lysates of B16F10 tumors, B16F10 cells, CT26 tumors, and CT26 cells (p-LKO, SHP2-silenced or cultured with 10 μm TNO155 for 72 hours) were measured by an antibody array with chemiluminescence detection (Mouse Angiogenesis Array Kit, R&D Systems, mLARY015). Tumor CCL2 levels were measured with the Mouse CCL2 DuoSet ELISA kit (R&D Systems, mLMJE00B), and the colorimetric reaction was measured at 450 nm using a microplate reader (Imgen Technologies, BMG LABTECH). These assays followed the manufacturer’s recommendations for sample preparation, procedures, and quantification. Western blotting was performed essentially as described before (11); the procedural details and antibodies used are listed in Supplemental Methods.

### Immunofluorescence, imaging, and image quantification.

Immediately after collection, tumor tissue samples were washed with cold PBS and fixed with cold 4% PFA for 72 hours at 4°C. Afterwards, tissues were processed by 24-hour incubations with PBS containing 10% (first), 20% (second) and 30% (third) sucrose and embedded in OCT compound (Sakura Finetek, mL4583). For immunostaining, 10 μm sections were layered onto glass slides and stored at –80°C. After thawing, sections were permeabilized with 1% Triton X-100/PBS (15 minutes), washed in PBS, and incubated with Uni-Trieve solution (Innovex Biosciences; mLNB325) at 65°C for 45 minutes. After washing 3 times with 1% Triton X-100/PBS, sections were blocked with blocking buffer (10% glycerol, 0.5% BSA, 0.4% Triton X-100, and 10% TBS) for 1 hour at room temperature. After rinsing in PBS, tissues were incubated overnight at 4°C with primary antibodies as detailed in Supplemental Methods. After the appropriate processing described in Supplemental Methods, secondary antibodies were used as detailed in Supplemental Methods. The slides were then washed 3 times with blocking buffer, postfixed with 4% PFA/PBS for 20 minutes at room temperature, washed 3 times with 1× TBS, and mounted with DAPI-containing mounting medium (MilliporeSigma, mLF6057). Images were obtained by confocal microscopy (LSM 780, LSM 880 or AxioScan Z1, Carl Zeiss) using ZEN software (Carl Zeiss). Image acquisition and quantification of fluorescently labeled markers are detailed in Supplemental Methods.

### Statistics.

For statistical analysis of the experimental results, no data were excluded from the analysis. The statistical significance of differences between 2 groups was calculated using the nonparametric Mann-Whitney *U* test or the parametric 2-tailed Student’s *t* test. Statistical significance among multiple groups was calculated by 1-way (with Tukey’s correction) or 2-way ANOVA (for multiple comparisons). *P* values of less than 0.05 were considered statistically significant. Analysis of the correlation between sets of data was performed using Spearman’s R. Data are presented as the mean or as means ± SEM.

### Study approval.

All animal experiments were approved by the Institutional Animal Care and Use Committee of the CCR, NCI, NIH and conducted in adherence with the NIH *Guide for the Care and Use of Laboratory Animals* (National Academies Press, 2011).

### Data availability.

Publicly available datasets of human cutaneous melanoma were downloaded (12/03/2023) from The Cancer Gene Atlas (TCGA), with the Project ID TCGA-SKCM. Datasets of human myelogenous leukemia MOLM13 cells control and after *INPPPL1* or *MAP4K5* gene KO are from the Gene Expression Omnibus (GEO) database (https://www.ncbi.nlm.nih.gov/geo/query/acc.cgi; GSE218491 and GSE212231). The Random-Forest object classifier based on object CD31 intensity and area (custom script written in Python) and the custom trained deep-learning Cellpose model (custom script written in Python) (85) are available at GitHub (https://github.com/CCRMicroscopyCore/wangy/releases/tag/wangy; commit ID: 65a32f9).

## Author contributions

YW and GT conceptualized the project, designed the experimental plan, and collected, analyzed, and interpreted the results. YW performed the animal treatment studies and performed in vitro experiments with contributions from HO, JXF, TH, MRS, RB, CL, and MS. MK and ADT performed image visualization and image quantification. HO, ESC, and DW performed bioinformatics data analyses. GT supervised the project. GT and YW wrote and edited the manuscript.

## Supplementary Material

Supplemental data

Unedited blot and gel images

Supporting data values

## Version 1 03/25/2025

In-Press Preview

## Version 2 05/15/2025

Electronic publication
