# Supplementary material for: Induced clustering of SHP2-depleted tumor cells in vascular islands restores sensitivity to MEK/ERK inhibition
Source: J Clin Invest. 2025 Mar 25;135(10):e181609. doi: 10.1172/JCI181609 (PMC12077907; doi:10.1172/JCI181609)
Supplement: Supplemental data [file jci-135-181609-s257.pdf]

SUPPLEMENTAL MATERIAL

Induced clustering of SHP2-depleted tumor cells in vascular islands restores sensitivity to MEK/ERK inhibition

Yuyi Wang, Hidetaka Ohnuki, Andy D. Tran, Dunrui Wang, Taekyu Ha, Jing-Xin Feng, Minji Sim, Raymond Barnhill, Claire Lugassy, Michael R. Sargen, Emanuel Salazar-Cavazos, Michael Kruhlak, and Giovanna Tosato

This file contains:

Methods

Supplemental Figure 1. Reduced tumor vascularization in SHP2-depleted B16F10 tumors.

Supplemental Figure 2. Signaling profiles of SHP2-depleted B16F10 cells from culture and SHP-2 depleted B16F10 tumors.

Supplemental Figure 3. Characterization of tumor islands in SHP2-silenced B16F10 tumors.

Supplemental Figure 4. Characterization of the vasculature in SHP2-silenced B16F10 tumors.

Supplemental Figure 5. Inhibition of VEGF/VEGFR signaling does not induce formation of tumor islands in B16F10 tumors.

Supplemental Figure 6. SHP2 depletion reduces vascular effector proteins in tumor cells.

Supplemental Figure 7. Cell components of B16F10 tumors and reduction of CCL2 levels in SHP2-silenced B16F10 tumors.

Supplemental Figure 8. SHP2 silencing in the murine colon carcinoma cell lines MC38 and CT26.

Supplemental Figure 9. PTPN11/SHP2 regulates expression of vascular factors in human cutaneous melanoma.

Supplemental Figure 10. The anti-tumor activity of GDC-0623 is magnified by SHP2 depletion in the tumor cells.

Supplemental Material

Methods

RNA purification, cDNA production, and real-time PCR. After RNA purification (RNeasy Micro Kit; QIAGEN 74004), cDNA was synthesized using High-Capacity cDNA Reverse Transcription kit (Applied Biosystems 4368814) and mRNA expression was measured by real-time PCR. Primers for mouse Ptpn11/Shp2 (F: 5'- GAAGTGGAGAGAGGGAAGAGCA-3'; R: 5'- CTGCCAGACGGTTCTCTCTGT-3'), mouse CCL2 (F: 5'- GCAGGTCCCTGTCATGCT TC -3'; R: 5'- TTCTTTGGGACACCTGCTGC -3'), mouse CCL3 (F: 5'- TCCCAGCCAGGTGTCATTTTCC-3'; R:5'-CACGGGACCCATGGAGGTTT-3'), mouse IL-1α (F: 5'- GTCGGGAGGAGACGACTCTAAA-3'; R: 5'- TGGTGTTTCTGGCAACTCCTTC-3') and GAPDH (F: 5'- GGCAAATTCAACGGCACAGT-3'; R: 5'-GGCGGAGATGATGACCCTTT-3') were designed using Primer3. Real-time PCR was run on a 7900HT Fast RT PCR System or QuantStudio™ 5 (Applied Biosystems) for 40 cycles with an annealing temperature of 60°C.

Flow cytometry and cell proliferation. To sort cell subpopulations from GFP^+ B16F10 tumors that had reached or approached 20mm in maximal diameter, single-cell suspensions were prepared by mincing tumor tissues into ~2 mm fragments, followed by addition of 2mg/ml collagenase diluted in PBS and incubation at 37°C for 1 hour. After dispersing the cells by gently pipetting and filtration through a cell strainer (70μm pores) to eliminate debris, cell suspensions were collected in a conical tube and washed twice in PBS containing 2% FBS. Cells were then incubated (30 minutes; room temperature) with BUV-737 anti-mouse VE-

cadherin antibody (BD Biosciences #741792) and PerCP/Cyanine5.5 anti-mouse CD45 Antibody (BD Biosciences #550994) and stained with propidium iodide (10μg/mg; Thermo Fisher Scientific, #P3566). Cell sorting was performed using FACSAriaIII-Fusion and flow cytometry analysis was performed on a SymphonyA5 (both instruments BD Biosciences). Results of flow cytometry were analyzed by FLOWJO software (v10.9.0, FlowJo LLC). To measure cell proliferation, 0.5 μCi 3H- thymidine (Perkin Elmer, #NET027WW0011MC) was added to 200 μL cell cultures in 96-well plates for 24 hours. Pulsed cells were collected from frozen (-80 °C) cultures onto glass fiber filters (Perkin Elmer, #1450-421), and incorporated radioactivity was counted in a liquid scintillation counter (Perkin Elmer; MicroBeta-1450 or MicroBeta-2450).

In-vitro island formation assay. Control p-LKO and SHP2-silenced B16F10 cells harvested from culture and washed twice with DMEM (no serum), were suspended ($8x10^5$ cells/40 μl) in DMEM (no serum) and kept on ice. The B16F10 cell suspension was mixed with equal volume Corning Matrigel (Scientific Laboratory Supplies LTD, #CLS354262; diluted 1:2 in ice-cold DMEM, no serum) to achieve a final cell concentration: $4x10^5$ cells/40 μl. The Secure-Seal Hybridization Chamber Gasket (Thermo Fisher, #S24732; Figure 3D) was attached to a cover glass (Ted Pella, Inc., #260460-100) and sterilized overnight by soaking in diluted (1:10 in PBS) Antibiotic-Antimycotic (Thermo Fisher, #15240062). After washing (3x in PBS) and air drying, the chamber was filled with the cold Matrigel-cell suspension (40 μl/chamber) and incubated 1 hour at 37°C to achieve Matrigel solidification. The chamber was then placed into a 10 cm dish containing 15 ml DMEM with 10% fetal bovine serum and 1x Antibiotic-Antimycotic. Since the chamber, made of polycarbonate plastic (250 μm thickness), is relatively gas-impermeable, gas

and medium exchange essentially occurs through the 2 holes present on the surface of the chamber (1.5 mm diameter, Figure 3D). The incubation time of cells dispersed into Matrigel inside the chamber ranged between 1-48 hours. The diffusion speed of biomolecules through the cell-free solidified Matrigel-filled chamber was analyzed by applying 3μl Alexa Fluor-594 conjugated Fab fragment of IgG (~50 kDa, Thermo Fisher #Z25307) to the chamber holes, and visualization of fluorescence (Olympus IX51) at time-intervals (Figure 3E). Propidium iodide (PI) staining (Figure 3G and H) of Matrigel-embedded cells in the chamber, was achieved by adding PI (0.5 mg/ml) to the gel and medium outside the chamber for 48 hours (1).

Images from inverted fluorescence microscopy (Olympus IX51). Quantification of PI^+ cells was performed using ImageJ. Immunostaining of islands formed *in vitro* after 48-hour culture (Figure 3I and J) was performed by first fixing cells and the Matrigel with 1% paraformaldehyde/PBS at room temperature overnight. Then, after soaking the chamber into 1x Tris-buffered saline (TBS) for 3 hours, followed by further soaking into blocking buffer (10% FBS/1% Triton X-100/1 mM EDTA/1x TBS) for 3 days with gentle agitation on a shaker at room temperature, the cells were stained with anti-Ki67 antibody labeled with Alexa Fluor 647 (Cell Signaling Technology, clone D3B5, #12075; 1:400 dilution in blocking buffer) for 4 days at 4°C with gentle agitation. Afterwards, the cells in the chamber were washed with blocking buffer for 1 day, and the anti-Ki67 rabbit antibody was blocked with high-affinity anti-rabbit IgG nanobody [1: 1000 dilution, clones CTK0101 (KD=0.2 nM) and CTK0102 (KD=1.2 nM), #srbAF488-1, ProteinTech] diluted with 2.5% BSA/1% Triton X-100/1mM EDTA/1x TBS for 2 days at 4°C. Cells in the chamber were then washed 3x with 1% Triton X-100/1 mM EDTA/1x PBS for 3 hours and anti-Ki67 and the nanobodies were fixed with 1% PFA/PBS overnight. After fixation, cells were washed and blocked 3x with blocking buffer for 3 hours. Then, cells were stained with a mixture of anti-p-

Erk1/2 antibody (1:400 dilution, approximately 12.55 pmole, Cell Signaling Technology, #4370) and anti-rabbit IgG nanobody labeled with Alexa Fluor 568 (50.2 pmole, clones CTK0101 and CTK0102, #srbAF568-1, ProteinTech) for 4 days. The anti-p-Erk1/2 and anti-rabbit IgG nanobodies were pre-incubated for 1 hour at room temperature before applying the chamber. Thereafter, cells were washed 3x with 1% Triton X-100/1 mM EDTA/1x PBS for 3 hours. The chamber was soaked with VectaShield containing DAPI (Vector Laboratories, #H-1000-10) diluted with 1 volume of TBS. After overnight incubation, the polycarbonate plastic side of the chamber was immobilized to a glass slide using a ProLong Diamond antifade mount (Thermo Fisher , #P36965). Images through the glass cover were taken by a Nikon SoRa Splining disc confocal microscope.

Western blotting. Cells harvested from culture by scraping were washed with cold phosphate buffered saline (PBS) containing 100nM sodium orthovanadate twice and lysed in TNTG buffer [(50mM Tris-HCL (pH 7.4), 150mM NaCl, 1% Triton X-100, 10% glycerol, 1×phosphatase inhibitor cocktail 2 (Sigma, #P5726) and 1×protease inhibitor cocktail (Thermo Scientific, #78430). Tumor lysates were prepared by placing 20-50 mg tissue into a sterile microcentrifuge capped tube (Bio Plas, Inc., #4204) containing a sterile, stainless-steel bead (7mm, Qiagen, #69990) and 0.5-1.0 ml TNGT buffer. To disrupt tumor tissue, the tubes were placed in Qiagen TissueLiser LT (Qiagen, #85600) at 50Hz, 5 min. Tumor lysates were centrifuged (10,000 g, 15 min) and supernatants stored at -80°C. Protein lysates were separated by SDS/PAGE using NuPage 4-12% Bis- Tris gels (Thermo Fisher, #NP0321) with either MOPS (Thermo Fisher, #NP0001) or MES (Crystalgen, #221110) running buffer and transferred to nitrocellulose membranes with iBot Gel Transfer system (Thermo Fisher, #IB301001 or #IB301002). After

blocking with 5% bovine albumin fraction V (MP Biomedicals, #160069) for 90 mins, membranes were incubated with primary antibodies: from Cell Signaling Technologies, SHP2 (#3397; 1:1000), p-STAT3 (Tyr^{705}) (#9145; 1:2,000); p-STAT3(Ser^{727})(#9134; 1:1000); STAT3 (#12640; 1;1000); p-Erk(Thr^{202}/Tyr^{204}) (#9101; 1:1,000); Erk (p44/p42) (#4695; 1:1,000); p-AKT (Ser^{473}) (#4060; 1:2,000), AKT (#9272; 1:1,000), GAPDH (5174; 1:1,000); from Santa Cruz Biotechnology, β-actin (#sc-47778; 1:1,000); from Novus Biologicals, CCL2/MCP1 (#NBP1-07035; 1:1000). HRP-conjugated secondary antibodies used: donkey anti-rabbit IgG (#NA934V; 1:5,000) and sheep anti-mouse IgG-Fc (#NA931V; 1:5,000) (both from GE Healthcare Life Sciences). Bands were visualized using ECL prime kit (GE Healthcare Life Sciences, #RPN2232) and captured digitally on a LAS4000 (GE LifeSciences) or Amersham Imager 680 (GE Healthcare). Protein bands from Western blotting were quantified by FIJI.

Immunofluorescence, imaging, and image quantification. Primary and secondary antibodies for immunofluorescence staining of tissue specimens and their use is described below. Rat anti-mouse CD31/PECAM (BD Pharmingen, #553370;1:100), rabbit anti-mouse Ki67 (Cell Signaling Technologies, #9129; 1:100), Alexa Fluor 647 rabbit anti-mouse Ki67 (Cell Signaling Technologies, #12075; 1:100), rabbit-anti-mouse pErk (Cell Signaling Technologies, #9101; 1:100), Rabbit-anti-mouse pCDK2 (Cell Signaling Technologies, #2561; 1:100), rabbit-anti-mouse SHP2 (Cell Signaling Technologies, #3397; 1:100), rabbit anti-Angiopoietin 2/Ang2 (Abcam, #8452; 1:200); rabbit anti-Cleaved caspase-3 (Cell Signaling Technologies, #9579; 1:100), rabbit anti-mouse collagen IV (Abd Serotec, #2150-1470; 1:100), rabbit anti-mouse α-SMA (Cell Signaling Technologies, #19245; 1:100), rabbit anti-mouse NG2 (Millipore, #AB5320; 1:100), rabbit anti-mouse Laminin (Millipore, #L9393, 1:200), Alexa Fluor 488 anti-

mouse CD45 (Biolegend, #103108, 1:100), Alexa Fluor 647 anti-mouse CD45 (Biolegend, #103124, 1:100), Alexa Fluor 488 anti-mouse F4/80 (Biolegend, #123120, 1:100), Alexa Fluor 647 anti-mouse F4/80 (Biolegend, #123121, 1:100), rat anti-mouse CD19 (BD Pharmingen, #553783;1:100) or rabbit anti-mouse CD3 (Abcam, #ab16669; 1:100). Slides were then washed (three times with blocking buffer). When needed, the following secondary antibodies were used: Alexa Fluor 488 donkey anti-rabbit IgG (Thermo Fisher; #A21206), Alexa Fluor 594 donkey anti-rat IgG (ThermoFisher; #A21209), Alexa Fluor 647 anti-rat IgG (ThermoFisher, #A-21247), Alexa Fluor 647 anti-rabbit IgG (ThermoFisher; #A-31573) and Alexa Fluor 546 donkey anti-rabbit IgG (ThermoFisher; #A-10040) for 1 h, at 4°C. The slides were then washed three times with blocking buffer, post-fixed with 4% PFA/PBS for 20 min at room temperature, washed three times with 1×TBS, and mounted with DAPI-containing mounting medium (Sigma, #F6057).

For image acquisition, extended field of view tile images of tissue sections were acquired using either the Zeiss LSM780 or LSM 880 laser scanning confocal microscope equipped with a 20× plan-apochromat (Numerical Aperture 0.8) objective lens and 32-channel gallium arsenide phosphide (GaAsP) spectral detector. Three or four fluorescence emission channels for the respective sample labels, DAPI (em 411-482), Alexa Fluor 488 (em 490-552), Alexa Fluor 561 (em 579-632 nm) and Alexa Fluor 633 (em 650-735 nm) were acquired. Tumor tissues and areas to be imaged were chosen randomly by operator. Confocal tile images were acquired with 0.277-0.519 µm (LSM780) 08-0.83 µm (LSM880) x-y pixel size and 5% image overlap.

For the quantification of fluorescently labeled markers in each unit tumor area, scanned images were exported as individual tiles. Autofluorescence from erythrocytes was removed

from each tile with freehand selection tool. The fluorescence from individual channels was then masked by a custom threshold. The size of masked fluorescent area was then measured in the individual tiles as a ratio of tile size. When noted, we also measured the ratio between two marker fluorescence in individual tiles.

For the quantification of marker fluorescence in individual tumor islands or sections, CD31 vessels were first segmented using a custom-trained *Random-Forest* machine learning pixel classifier (*scikit-learn*) based on original fluorescence intensities, various kernel-sized Median and entropy convolutions and various Gabor filters (2).

The subsequent foreground pixels were grouped into objects using connected components analysis, and the objects were filtered using an independent *Random-Forest* object classifier based on object CD31 intensity and area (custom script written in Python available upon request). CD31-islands were further defined by extending the borders of the CD31-positive vessels by 250 pixels. These segments were used as Regions Of Interest (ROIs) for intensity quantifications of individual cell markers. For the quantification of fluorescence in individual cells, single cells were segmented using a custom trained deep-learning Cellpose model (custom script available here: https://github.com/CCRMicroscopyCore/wangy

Additional references pertinent to the methods

1. Zhao H, Oczos J, Janowski P, Trembecka D, Dobrucki J, Darzynkiewicz Z, and Wlodkowic D. Rationale for the real-time and dynamic cell death assays using propidium iodide. *Cytometry A.* 2010;77(4):399-405.

2. Pedregosa F, Varoquaux G, Gramfort A, Michel V, Thirion B, Grisel O, et al. Scikit-learn: Machine Learning in Python. *Journal of Machine Learning Research.* 2011;12:2825-30.

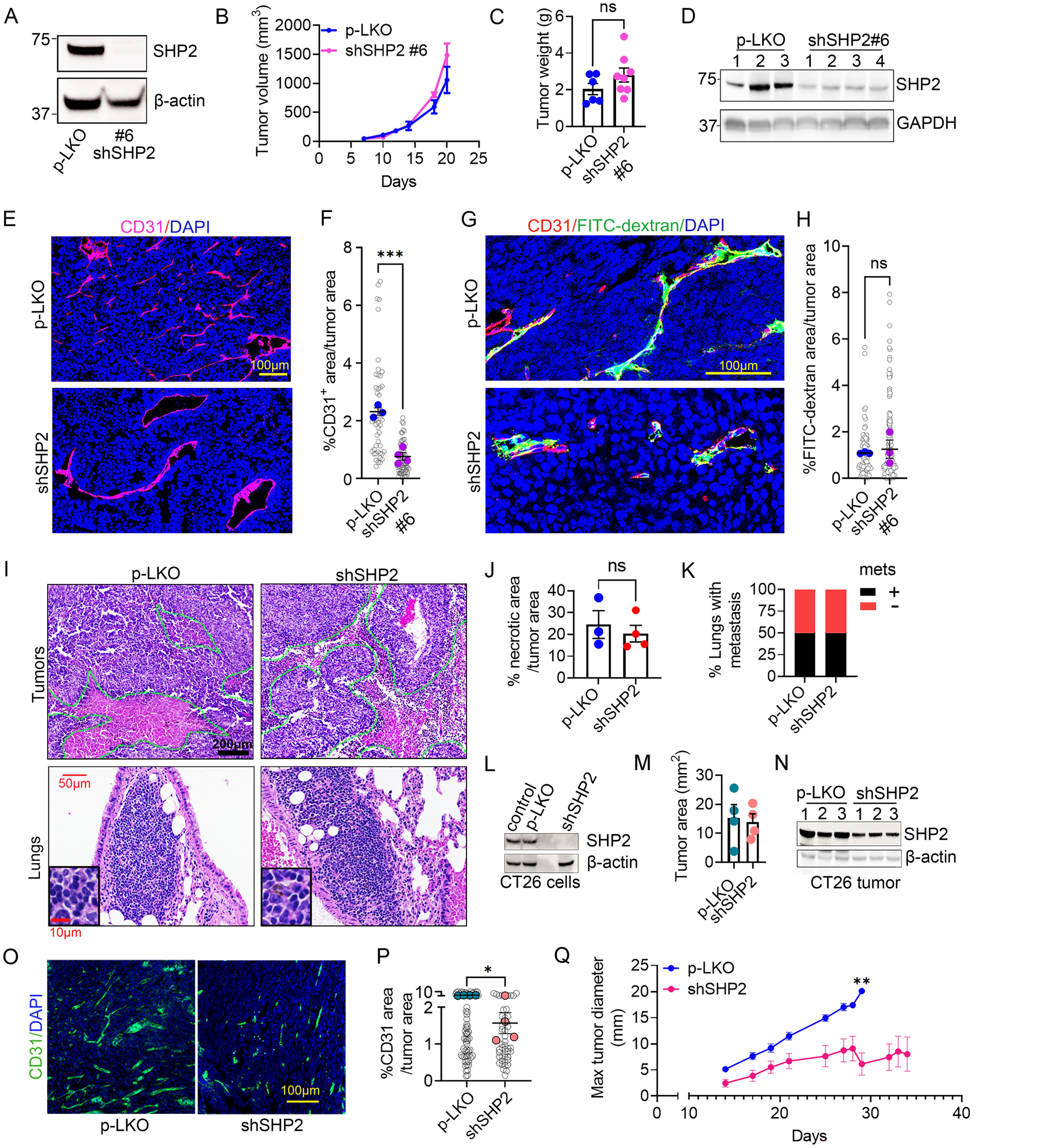

Supplemental Figure 1. Reduced tumor vascularization in SHP2-depleted B16F10 tumors. (**A**) SHP2 depletion from B16F10 cells by shRNA#6. (**B, C**) B16F10 cells (1×10^6) control (p-LKO) or SHP2-silenced (shRNA#6) were inoculated s.c. into groups of mice: control no. 6; SHP2-silenced no. 8. Tumor volume (B) and tumor weight at the 21-day endpoint (C). ns: not significantly different by two-tailed Student's t-test. (**D**) SHP2 is depleted in SHP2-silenced B16F10 tumors at harvest (shSHP2#6). (**E, F**) $CD31^+$ blood vessels are reduced in SHP2-silenced (shSHP2#6) B16F10 tumors compared to control (p-LKO). Representative images (E) and

image quantification (F); each grey circle represents the % $CD31^+$ area/unit tumor area; each colored dot represents the mean % $CD31^+$ area/tumor (p-LKO n=3; shSHP2 n=4). ***P<0.001 by two-tailed Student's *t*-test (**G, H**). Vessel perfusion by FITC-dextran in control (p-LKO) and SHP2-silenced (shSHP2#2) B16F10 tumors. Representative images (G). Quantification of FITC-dextran pixel area/unit tumor area; each grey circle represents the % FITC-$dextran^+$ area/unit tumor area; each colored dot represents the mean % FITC-$dextran^+$ area/tumor (p-LKO n=3; shSHP2 n=3) (H). ns: not significant by two-tailed Student's *t*-test. (**I**) Representative images of H&E-stained primary and metastatic (to lung) B16F10 melanoma. (**J**) Tumor tissue necrosis quantified in control (p-LKO; n=3) and SHP2-silenced (shSHP2#2; n=4) B16F10 tumors (day 21 post inoculation). The green outlines demark dead and live tissue from H&E-stained tumor tissues. Not significant (ns) by two-tailed Student's t-test. (**K**) % lungs (n=6/group) with or without metastases (mets) in mice bearing control (p-LKO) and SHP2-silenced (shSHP2#6) B16F10 primary tumors (21-day endpoint). (**L-N**) SHP2-silenced and p-LKO control CT26 colon carcinoma cells (L) inoculated s.c. into syngeneic mice (no. 4/group) gave rise to similar-sized tumors (M); tumors from SHP2-silenced CT26 cells displayed reduced SHP2 compared to control (N) at harvest. (**O, P**) Vascularization of SHP2-silenced CT26 tumors (n=4) is reduced compared controls (n=4). Representative images (O) and quantification; each grey circle represents the % $CD31^+$ area/unit tumor area; each colored dot represents the mean %$CD31^+$ area/tumor (P). *P<0.05 by two-tailed Student's t-test. (**Q**) Tumor growth from s.c. inoculation of SHP2-silenced (shSHP2#1, #2 or #3; n=15) or control (p-LKO n=5) B16F10 cells (2.5×10^4). **P<0.01 on day 28 post inoculation by two-tailed Student's t-test.

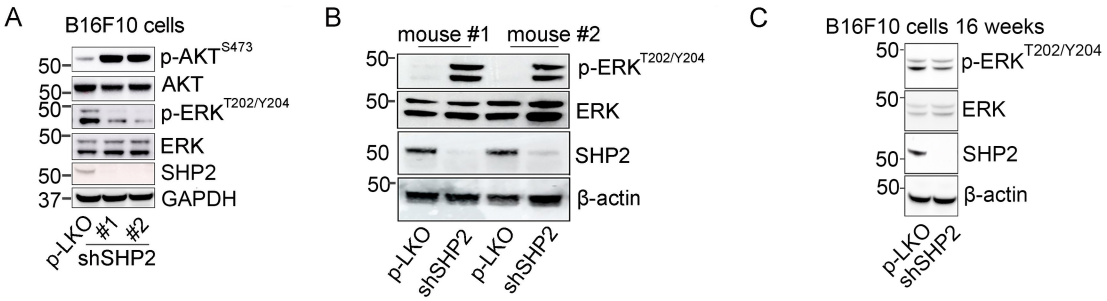

Supplemental Figure 2. Signaling profiles of SHP2-depleted B16F10 cells from culture and SHP-2 depleted B16F10 tumors. (A) Activity of signaling molecules in control and SHP2-silenced (shSHP2#1 and #2) B16F10 cells propagated in culture. Immunoblotting results. **(B)** Lysates from control and SHP2-depleted B16F10 tumors were evaluated for p-ERK$^{T202/Y204}$ by immunoblotting. **(C)** Signaling profile of SHP2-silenced B16F10 cells evaluated after culture in vitro for 16 weeks after silencing.

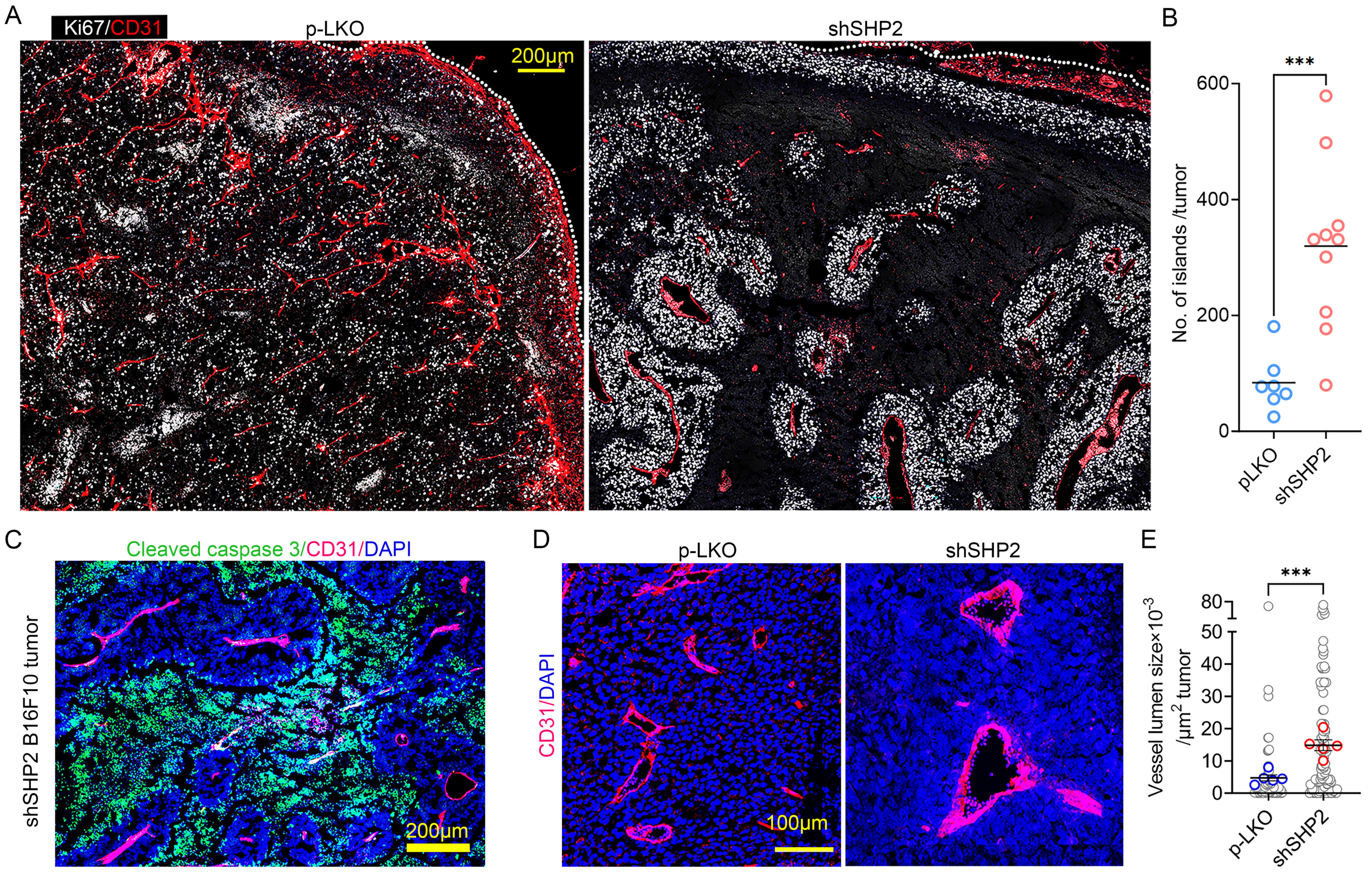

Supplemental Figure 3. Characterization of tumor islands in SHP2-silenced B16F10 tumors. (**A, B**) Ki67 and CD31 immunostaining in control and SHP2-silenced B16F10 tumors (harvest day 21). Representative confocal microscopy images (A) and quantification of island-like structures (B) in control and SHP2-silenced B16F10 tumors. For quantification, an island is defined as live tumor tissue area ($Ki67^+$) surrounding a blood vessel ($CD31^+$) generally limited by $Ki67^-$ tumor. Quantification performed by evaluation of entire tumor sections through the maximum diameter; individual dots reflect quantification of separate tumors (control n=7; SHP2-silenced n=10). ***P<0.001 by two-tailed Student's t-test. (**C**) Cleaved caspase-3 immunostaining identifies dead B16F10 tumor tissue generally surrounding tumor islands composed of DAPI+ tumor cells centered by $CD31^+$ blood vessels; representative image (tumor harvest day 21). (**D, E**) Representative blood vessels ($CD31^+$) from control and SHP2-silenced B16F10 tumor tissues (D); vessel caliber (lumen size; E); each grey circle represents vessel caliber/unit tissue area; each blue and red circle reflects the mean vessel caliber/tumor (control: 5 tumors; SHP2-silenced: 5 tumors). ***P<0.001 by Student's *t*-test.

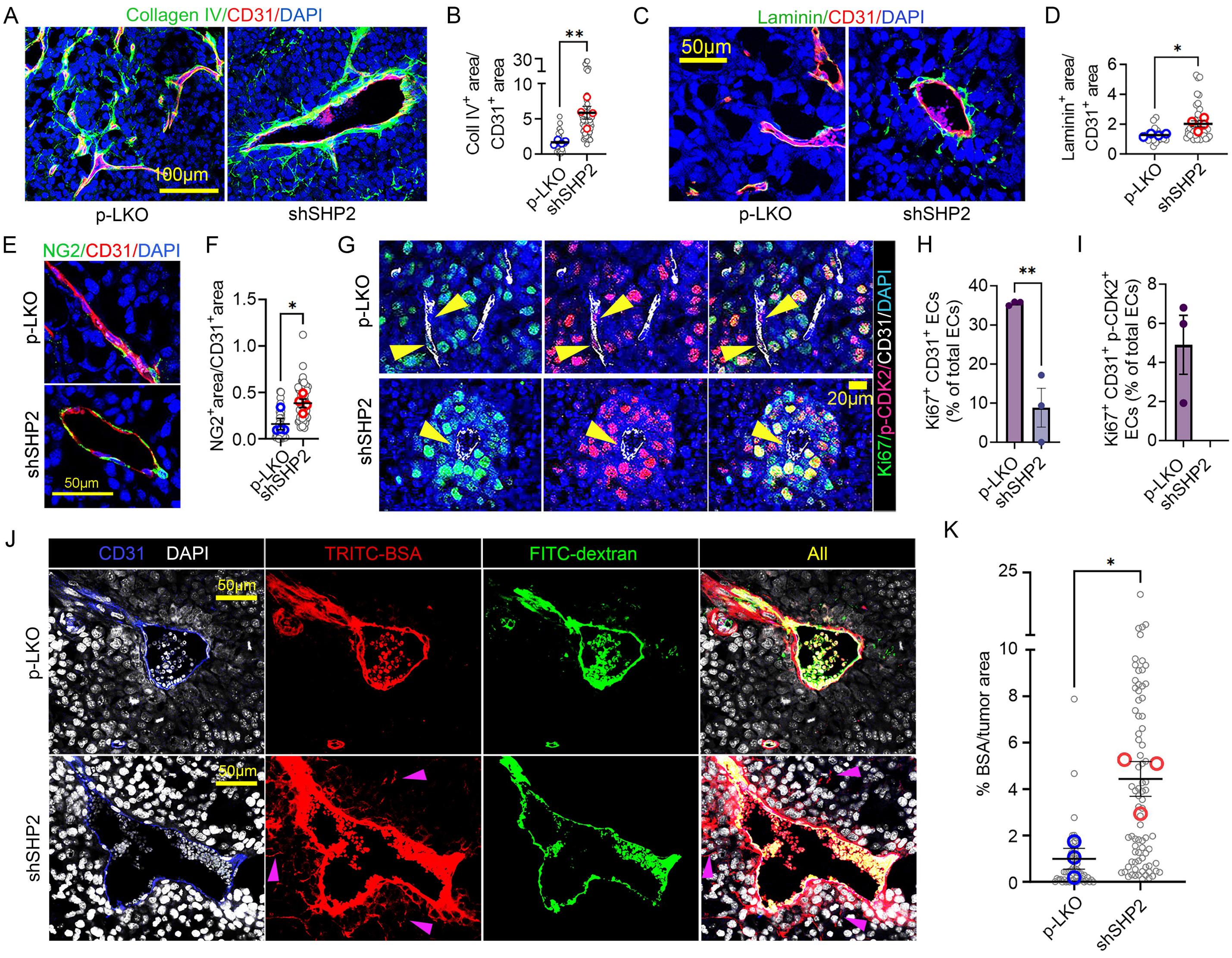

Supplemental Figure 4. Characterization of the vasculature in SHP2-silenced B16F10 tumors. (A, B) Collagen IV immunostaining in representative control and SHP2-silenced B16F10 tumors (A); quantification of Collagen IV immunostaining associated with $CD31^+$ blood vessels in control (n=4) and SHP2-silenced B16F10 tumors (n=4). Each grey circle represents the Collagen IV^+ area/$CD31^+$ area/unit tumor area; each blue and red circle represents the tumor mean (B). **P<0.01 by two-tailed Student's *t*-test. (**C, D**) Laminin immunostaining of blood vessels ($CD31^+$) in representative tissue sections of control and SHP2-silenced B16F10 tumors (C); quantification of Laminin immunostaining associated with $CD31^+$ blood vessels in control (n=4) and SHP2-silenced B16F10 tumors (n= 4). Each grey circle represents the $Laminin^+$ area/$CD31^+$ area/unit tumor area; each blue and red circle represents the tumor mean (D). *P<0.05 by two-tailed Student's *t*-test. **(E, F)** NG2 immunostaining in representative control and SHP2-silenced B16F10 tumors (E); quantification of NG2 immunostaining associated with $CD31^+$ blood vessels in control (n=4) and SHP2-silenced B16F10 tumors

(n=4). Each grey circle represents the $NG2^+$ area/$CD31^+$ area/tumor unit area; each blue and red circle represents the tumor mean (F). *P<0.05 by two-tailed Student's *t*-test. (**G-I**) Representative images showing immunostaining of p-LKO and SHP2-silenced B16F10 tumors for KI67, p-CDK2, CD31 and DAPI (G); $Ki67^+$ (H) and $Ki67^+$p-$CDK2^+$ (I) $CD31^+$ endothelial cells are more frequent in control (n=3) than in shSHP2 B16F10 tumors (n=3). **P<0.001 by two-tailed Student's t-test. (**J, K**) Vessel perfusion by Tetramethylrhodamine isothiocyanate-conjugated BSA (TRITC, red; molecular weight ~66 kDa) and FITC-dextran (2,000,000 MW) in control and SHP2-silenced B16F10 tumors (harvest day 21). Representative confocal microscopy images of FITC-dextran-perfused vessels showing penetration of TRITC-BSA in the SHP2-silenced tumor (pointed by the arrows) (J); quantification (K) of TRITC-BSA immunostaining in control (n=3) and SHP2-silenced B16F10 tumors (n= 3). Each grey circle represents the BSA^+ area/tumor unit that excludes the vessel area; each blue and red circle reflects the tumor mean. *P<0.05 by two-tailed Student's *t*-test.

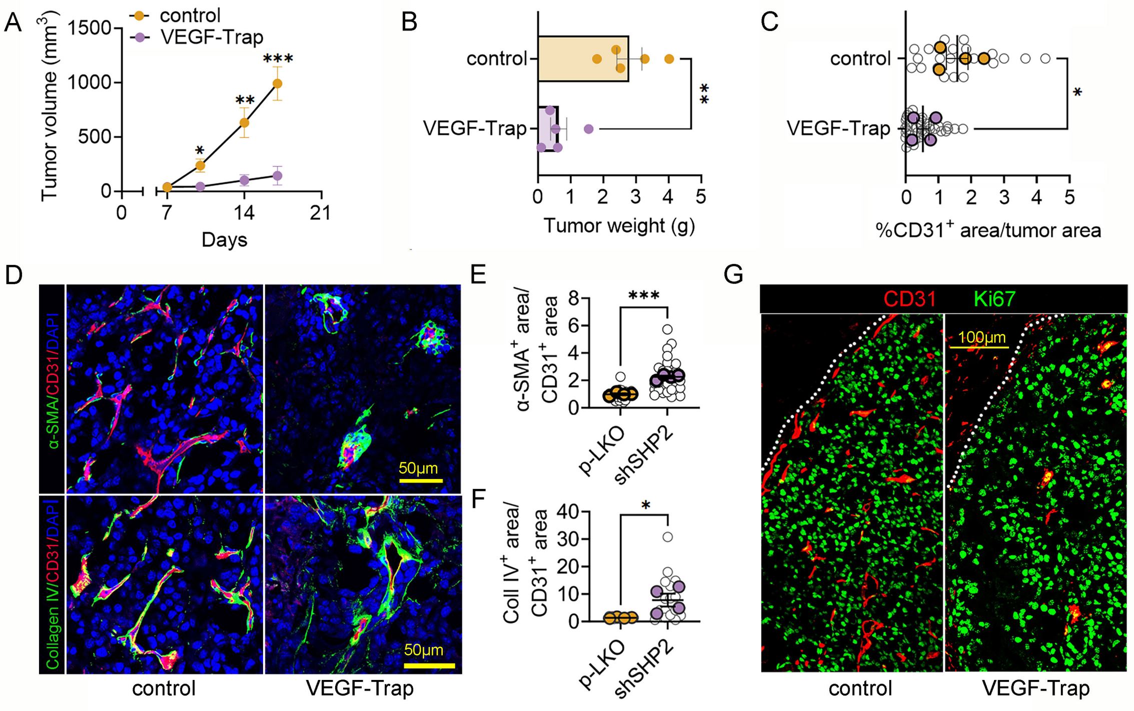

Supplemental Figure 5. Inhibition of VEGF/VEGFR signaling does not induce formation of tumor islands in B16F10 tumors. (**A**) B16F10 tumor-bearing mice (n=5/group) were treated with VEGF-Trap (5mg/kg) or buffer only. The experiment was terminated when the maximal tumor diameter reached 20 mm in any mouse. Tumor volume *P< 0.05, **P<0.01, ***P<0.001 by two-tailed Student's t-test. (**B**) Tumor weight at harvest; **P<0.01. **(C)** $CD31^+$ vessel quantification in B16F10 tumors treated with VEGF-Trap or buffer only; each grey circle reflects the % $CD31^+$ area/tumor unit area; the colored dots reflect the tumor mean (n=4/group). *P<0.05 from by two-tailed Student's *t*-test. (**D-F**) α-SMA and Collagen IV immunostaining in representative control and VEGF-Trap treated B16F10 tumors (D). Quantification of α-SMA/Collagen IV immunostaining associated with $CD31^+$ blood vessels in control (n=4) and VEGF-Trap treated tumors (n=4). In (E) each grey circle represents the α-SMA^+ area/$CD31^+$ area; the color dots reflect the tumor mean (n=4/group), and in (F) each grey circle represents the Collagen IV^+ area/$CD31^+$ area/unit tumor area; the colored dots reflect the tumor mean (n=4/group) (F). *P<005 ***P<0.001 by two-tailed Student's *t*-test. (**G**) Ki67 and CD31 immunostaining in representative control and VEGF-Trap B16F10 tumors.

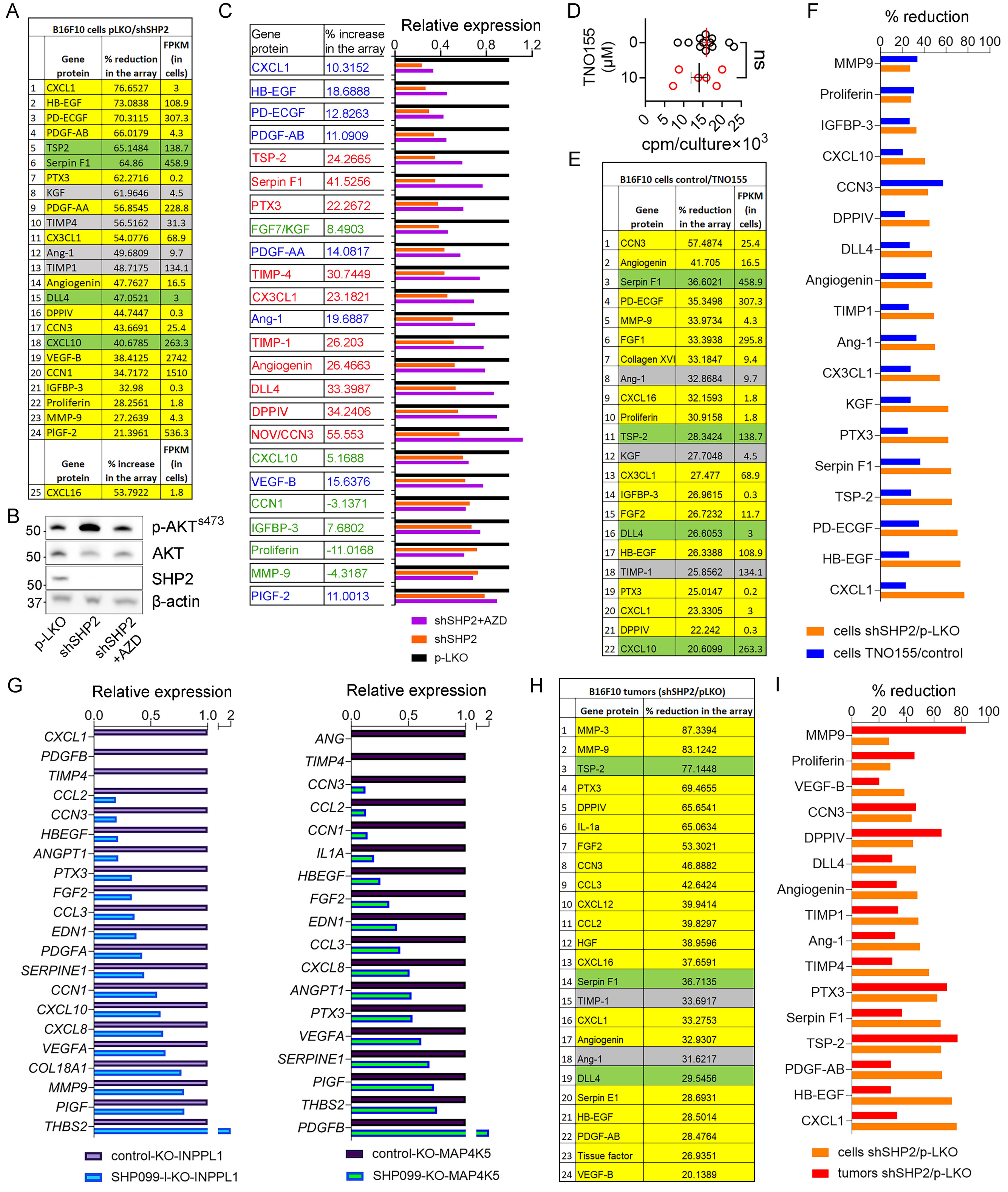

A — B16F10 cells pLKO/shSHP2

	Gene protein	% reduction in the array	FPKM (in cells)
1	CXCL1	76.6527	3
2	HB-EGF	73.0838	108.9
3	PD-ECGF	70.3115	307.3
4	PDGF-AB	66.0179	4.3
5	TSP2	65.1484	138.7
6	Serpin F1	64.86	458.9
7	PTX3	62.2716	0.2
8	KGF	61.9646	4.5
9	PDGF-AA	56.8545	228.8
10	TIMP4	56.5162	31.3
11	CX3CL1	54.0776	68.9
12	Ang-1	49.6809	9.7
13	TIMP1	48.7175	134.1
14	Angiogenin	47.7627	16.5
15	DLL4	47.0521	3
16	DPPIV	44.7447	0.3
17	CCN3	43.6691	25.4
18	CXCL10	40.6785	263.3
19	VEGF-B	38.4125	2742
20	CCN1	34.7172	1510
21	IGFBP-3	32.98	0.3
22	Proliferin	28.2561	1.8
23	MMP-9	27.2639	4.3
24	PIGF-2	21.3961	536.3
	Gene protein	% increase in the array	FPKM (in cells)
25	CXCL16	53.7922	1.8

C

Gene protein	% increase in the array
CXCL1	10.3152
HB-EGF	18.6888
PD-ECGF	12.8263
PDGF-AB	11.0909
TSP-2	24.2665
Serpin F1	41.5256
PTX3	22.2672
FGF7/KGF	8.4903
PDGF-AA	14.0817
TIMP-4	30.7449
CX3CL1	23.1821
Ang-1	19.6887
TIMP-1	26.203
Angiogenin	26.4663
DLL4	33.3987
DPPIV	34.2406
NOV/CCN3	55.553
CXCL10	5.1688
VEGF-B	15.6376
CCN1	-3.1371
IGFBP-3	7.6802
Proliferin	-11.0168
MMP-9	-4.3187
PIGF-2	11.0013

E — B16F10 cells control/TNO155

	Gene protein	% reduction in the array	FPKM (in cells)
1	CCN3	57.4874	25.4
2	Angiogenin	41.705	16.5
3	Serpin F1	36.6021	458.9
4	PD-ECGF	35.3498	307.3
5	MMP-9	33.9734	4.3
6	FGF1	33.3938	295.8
7	Collagen XVI	33.1847	9.4
8	Ang-1	32.8684	9.7
9	CXCL16	32.1593	1.8
10	Proliferin	30.9158	1.8
11	TSP-2	28.3424	138.7
12	KGF	27.7048	4.5
13	CX3CL1	27.477	68.9
14	IGFBP-3	26.9615	0.3
15	FGF2	26.7232	11.7
16	DLL4	26.6053	3
17	HB-EGF	26.3388	108.9
18	TIMP-1	25.8562	134.1
19	PTX3	25.0147	0.2
20	CXCL1	23.3305	3
21	DPPIV	22.242	0.3
22	CXCL10	20.6099	263.3

H — B16F10 tumors (shSHP2/pLKO)

	Gene protein	% reduction in the array
1	MMP-3	87.3394
2	MMP-9	83.1242
3	TSP-2	77.1448
4	PTX3	69.4655
5	DPPIV	65.6541
6	IL-1a	65.0634
7	FGF2	53.3021
8	CCN3	46.8882
9	CCL3	42.6424
10	CXCL12	39.9414
11	CCL2	39.8297
12	HGF	38.9596
13	CXCL16	37.6591
14	Serpin F1	36.7135
15	TIMP-1	33.6917
16	CXCL1	33.2753
17	Angiogenin	32.9307
18	Ang-1	31.6217
19	DLL4	29.5456
20	Serpin E1	28.6931
21	HB-EGF	28.5014
22	PDGF-AB	28.4764
23	Tissue factor	26.9351
24	VEGF-B	20.1389

Supplemental Figure 6

Supplemental Figure 6. SHP2 depletion reduces vascular effector proteins in tumor cells. (**A**) Angiogenic regulators reduced >20% in SHP2-silenced B16F10 cells compared to controls; % protein reduction shown. mRNA expression levels in B16F10 cells (Fragments/Kilobase Per Million mapped fragments (FKPM)); RNAseq data: GSE212112. Yellow denotes pro-angiogenic proteins; green denotes anti-angiogenic proteins; grey denotes context-dependent activity. **(B)** The p-AKT inhibitor AZD2014 (0.1µM) reduces p-AKT^{S473} in SHP2-silenced B16F10 cells; 3-day culture; immunoblotting results. (**C**) Relative reduction of angiogenesis-related proteins by SHP2 silencing in B16F10 cells (red bars) and effects of AZD2014 on this inhibition (purple bars); protein levels observed in p-LKO B16F10 are set at 100% (black bars). Increase >20: red; >10: blue; other change: green. **(D)** TNO155 (10µM) minimally alters the *in vitro* proliferation of B16F10 cells. (**E**) List of angiogenesis-related proteins reduced >20% in TNO-treated B16F10 cells compared to controls. Degree of protein reduction is shown. **(F)** Relative reduction of angiogenesis-related proteins induced by SHP2 silencing and TNO155 treatment of B16F10 cells *in vitro*. **(G)** Reduced expression of angiogenesis-related genes in *INPPL1* (left) and *MAP4K5* (right) knockout MOLM13 cells treated *in vitro* with SHP099; RNAseq results (GSE218491; GSE212231) are expressed as relative expression. (**H**) Relative reduction of angiogenesis-related proteins in B16F10 tumors from inoculation of SHP2-silenced B16F10 cells or p-LKO B16F10 cells. (**I**) Relative reduction of angiogenesis-related proteins in SHP2-silenced compared to control B16F10 cells *in vitro* and in SHP2-silenced B16F10 tumors compared to control tumors.

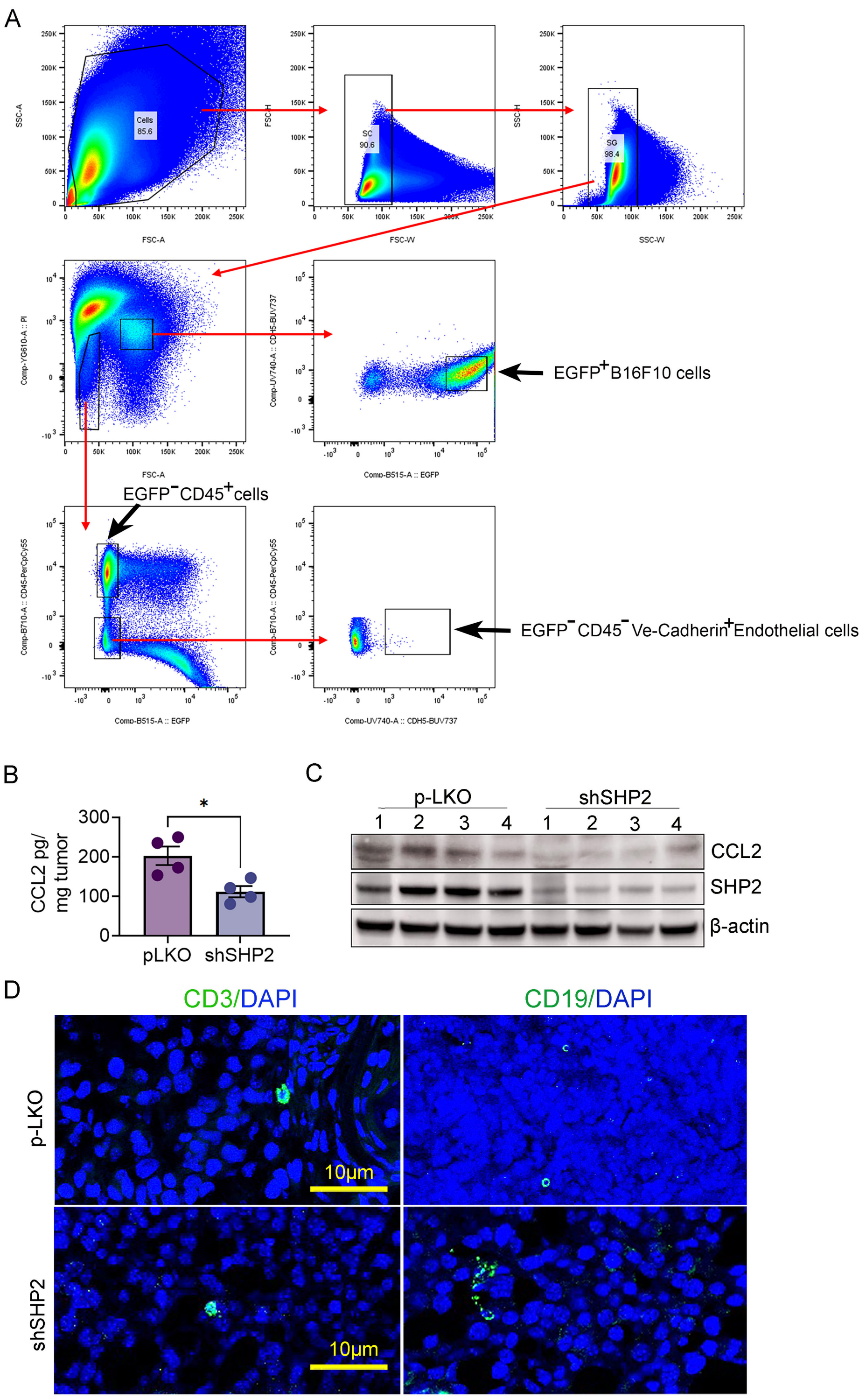

Supplemental Figure 7

Supplemental Figure 7. Cell components of B16F10 tumors and reduction of CCL2 levels in SHP2-silenced B16F10 tumors. (A) Cell sort of GFP^+ B16F10 tumor cells, GFP^-CD45^+ cells and GFP^-CD45^-VE-Carherin$^+$ cells from single-cell suspensions of B16F10 tumors generated by s.c. inoculation of GFP^+ B16F10 tumor cells. (**B, C**) CCL2 protein in lysates of SHP2-silenced (no. 4) and p-LKO control (no. 4) B16F10 tumors measured by ELISA (B) and immunoblotting (C). *$P<0.05$ by two-tailed Student's *t*-test. **(D)** Immunofluorescence detection of rare $CD3^+$ and $CD19^+$ lymphocytes in B16F10 tumor sections from p-LKO control and SHP2-silenced B16F10 tumors.

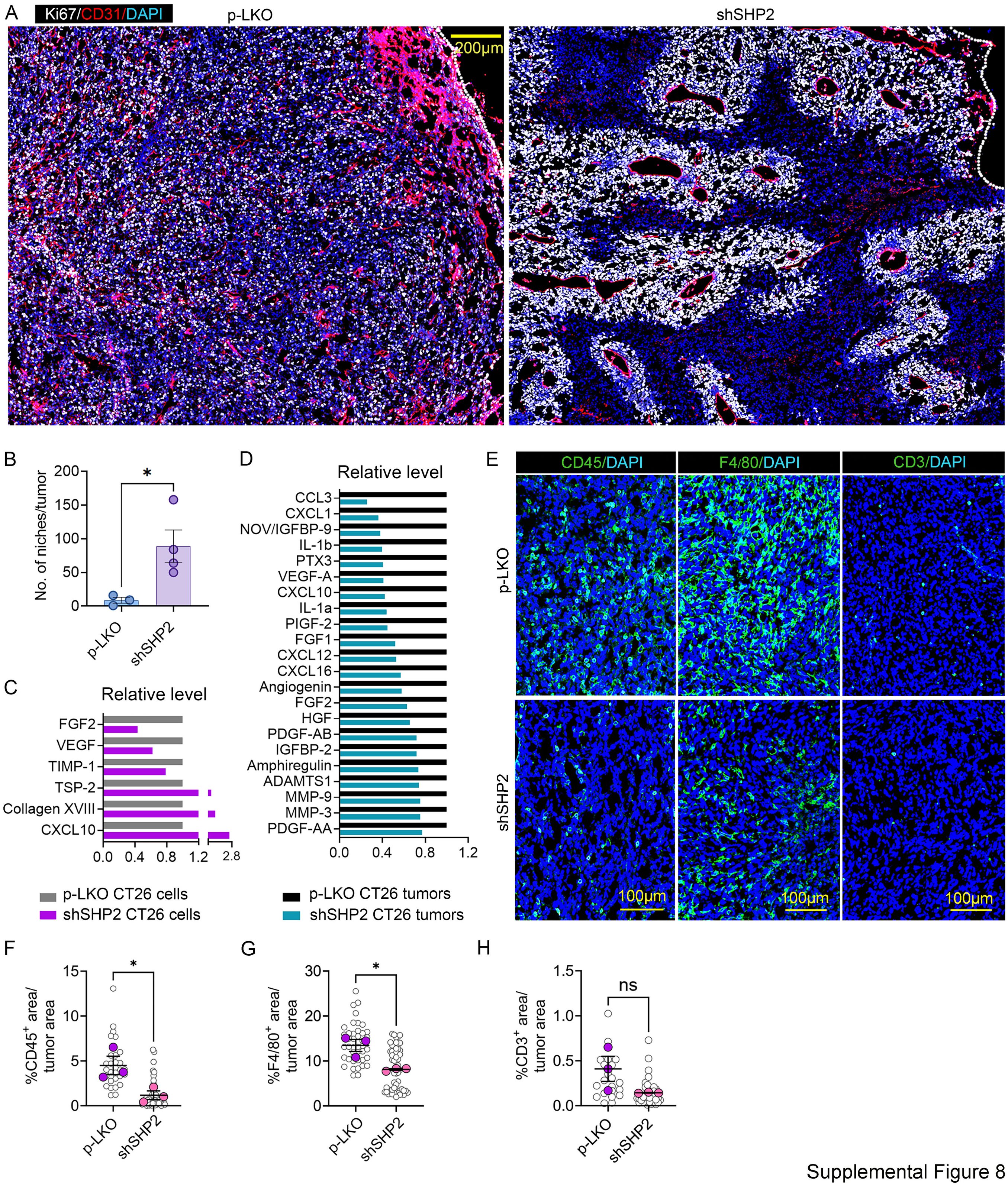

Supplemental Figure 8

Supplemental Figure 8. SHP2 silencing in the murine colon carcinoma cell lines MC38 and CT26. (**A** and **B**) Characteristic architecture of SHP2-silenced MC-38 tumors showing the clustering of $Ki67^{+}$ (white) tumor cells around $CD31^{+}$ blood vessels (red) separated by non-proliferative ($Ki67^{-}$) and poorly vascularized ($CD31^{-}$) tumor areas; representative confocal images from control and SHP2-silenced tumors (A) and quantification of tumor islands (B) in each of the tumors (no. 3/p-LKO group; no. 4/shSHP2 group). *P<0.05 from two-tailed Student's t-test. (**C** and **D**) Content of angiogenic and other factors in SHP2-silenced colon carcinoma CT26 cells from culture (D), and tumors from inoculation of SHP2-silenced or control CT26 (E) compared to controls. Lysates from SHP2-silenced (n=3) and p-LKO control (n=3) CT26 tumors were applied to the Proteome Profiler Array. (**E-H)** $CD45^{+}$ leukocytes (E and F), $F4/80^{+}$ macrophages (E and G), and $CD3^{+}$ lymphocytes (E and H) infiltrates in SHP2-silenced MC-38 tumors compared to control (p-LKO). Representative images (E) and quantification (F-H); each grey circle represents the % positive area/unit tumor area; the colored dots (n=3/group) reflect the tumor mean; *P<0.05 and ns, not significant by two-tailed Student's *t*-test.

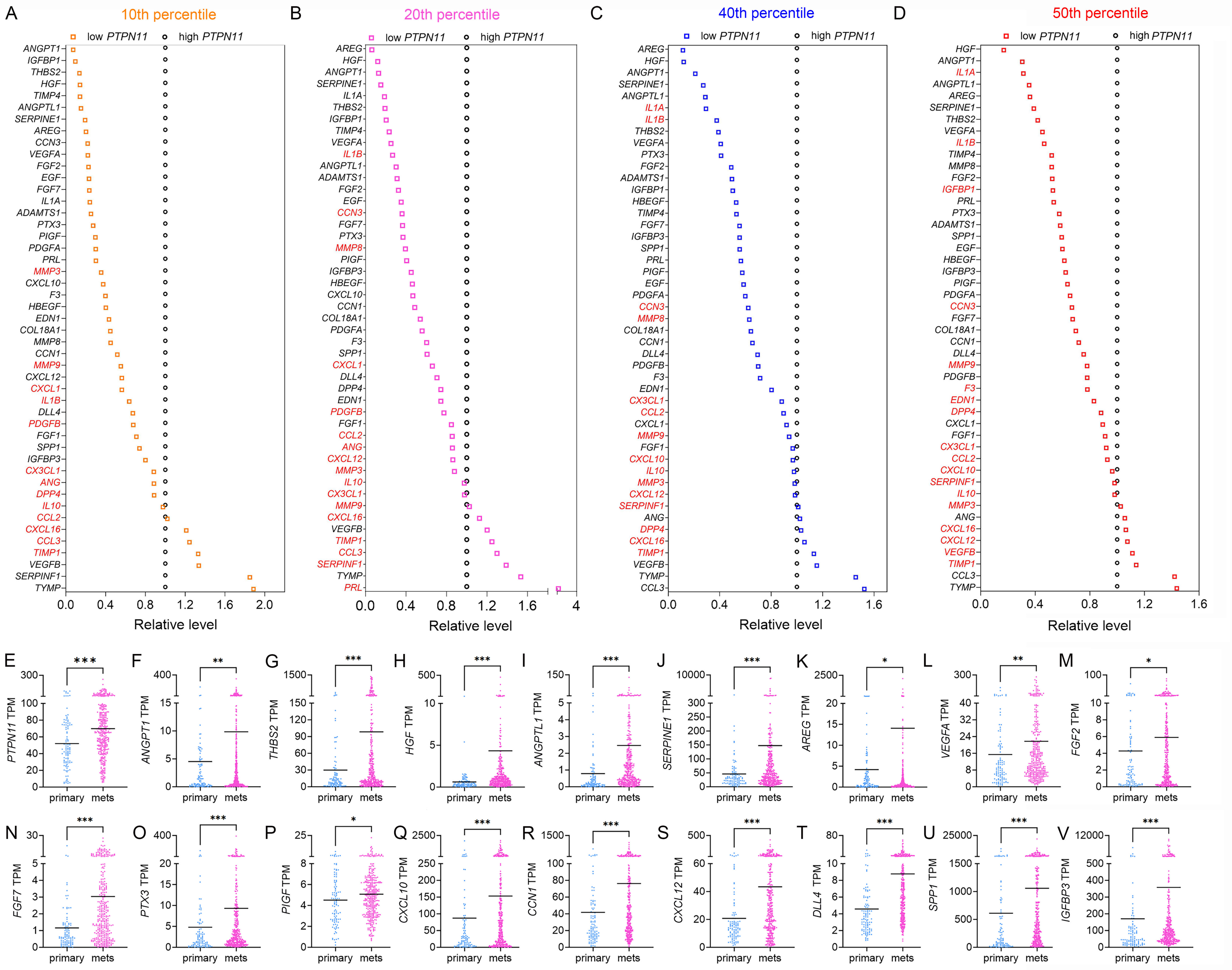

Supplemental Figure 9

Supplemental Figure 9. PTPN11/SHP2 regulates expression of vascular factors in human cutaneous melanoma. (A-D) Relative mRNA expression levels of the indicated genes in human cutaneous melanoma (TCGA-SKCM) expressing high or low PTPN11 levels. (A) 10% high and 10% low PTPN11 levels (no=47/group); (B) 20% high and 20% low PTPN11 levels (no=94/group); (C) 40% high and 40% low PTPN11 levels (no=188/group); and (D) 50% high or 50% low PTPN11 levels (no=236 low/no=235 high). Each dot represents the relative mean expression level; expression of the genes in red ink is not statistically different in the two groups by two-tailed Student's t test. **(E)** Expression levels of *PTPN11* are significantly higher in metastatic (mets; n=368) compared to primary (n=103) human melanoma (TCGA-SKCM). (**F-V**) Expression levels of the indicated factors in metastatic (n=368) compared to primary (N=103) human melanoma (TCGA-SKCM). Results in E-V are expressed as TPM (transcripts per million reads). *$P<0.05$, **$P<0.01$, ***$P<0.001$, by Mann-Whitney U test.

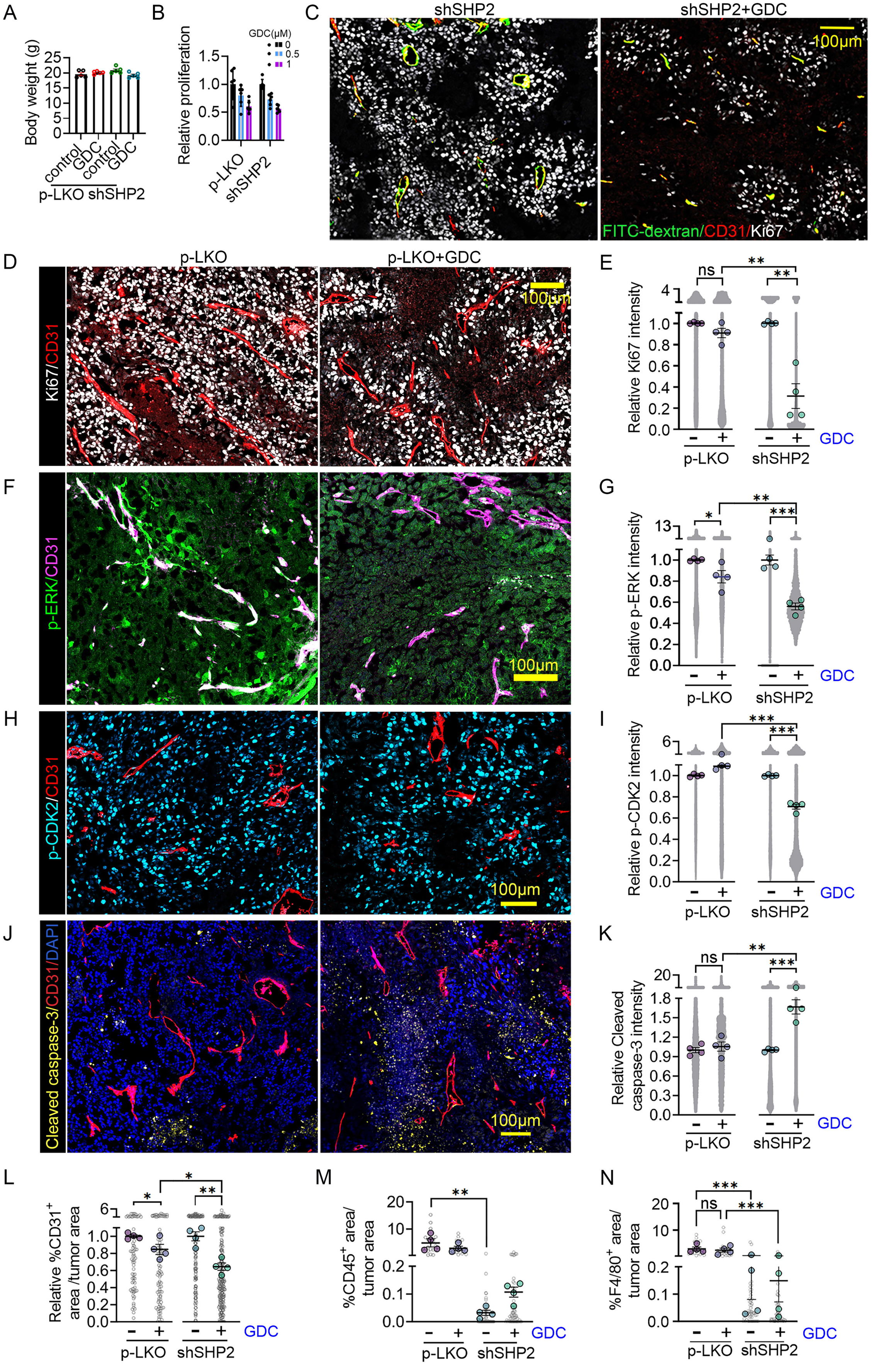

Supplemental Figure 10

Supplemental Figure 10. The anti-tumor activity of GDC-0623 is magnified by SHP2 depletion in the tumor cells. (**A**) Body weight of mice with B16F10 tumors (p-LKO or shSHP2) after treatment with GDC-0623 or buffer only. Results reflect means (± SEM); no. 5/group. (**B**) GDC-0623 dose-dependently reduces proliferation of SHP2-silenced and control (p-LKO) B16F10 cells. Results from ^{3}H thymidine incorporation after 72 hr incubation are displayed as relative proliferation/culture (± SD); n=6. (**C**) Vessel perfusion by FITC-dextran in SHP2-silenced B16F10 tumors treated with GDC-0623 or buffer only is shown in representative images. (**D-K**) Representative confocal images of control (p-LKO) B16F10 tumor tissues treated with GDC-0623 or buffer only (left) and immunostaining quantification (right) in the 4 treatment groups: control (p-LKO) and SHP2-silenced B16F10 tumors treated with GDC-0623 or buffer only. Quantitative results are displayed as relative fluorescence intensity of GDC-treated groups relative to the untreated p-LKO and shSHP2 groups. Ki67 (D, E); p-ERK1/2 (F, G); p-CDK2 (H, I), and Cleaved caspase-3 (J, K). The grey areas reflect accumulation of individual datapoints reflecting relative fluorescence intensity/unit tumor area; the colored circles reflect the tumor mean n=4 tumors/group). *P<0.05, **P<0.01, ***P<0.001 and ns, not significant by two tailed Student's *t*-test. The results in E, G, I, K and L (shSHP2 samples) are the same results shown in Figure 9 (K-O). (**L-N**) Quantification of CD31 (L), CD45 (M) and F4/80 (N) immunostaining in tumor tissues from the 4 treatment groups. The results are shown as relative % fluorescent area/unit tumor area (L) or % fluorescent area/tumor area (M and N) in the grey circles and tumor mean (colored dots). *P<0.05, **P<0.01 two tailed Student's *t*-test. (L) or **P<0.01, ***P<0.001 and ns, not significant by one-way ANOVA (M and N).
