# Supplementary material for: Induced clustering of SHP2-depleted tumor cells in vascular islands restores sensitivity to MEK/ERK inhibition
Source: J Clin Invest. 2025 Mar 25;135(10):e181609. doi: 10.1172/JCI181609 (PMC12077907; doi:10.1172/JCI181609)
Supplement: Unedited blot and gel images [file jci-135-181609-s258.pdf]

Figure 1

Panel A

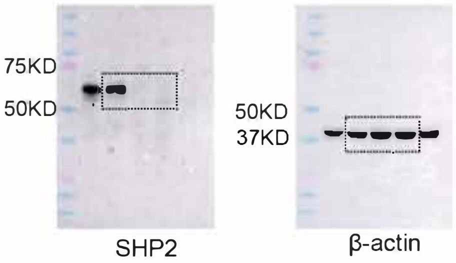

Panel D

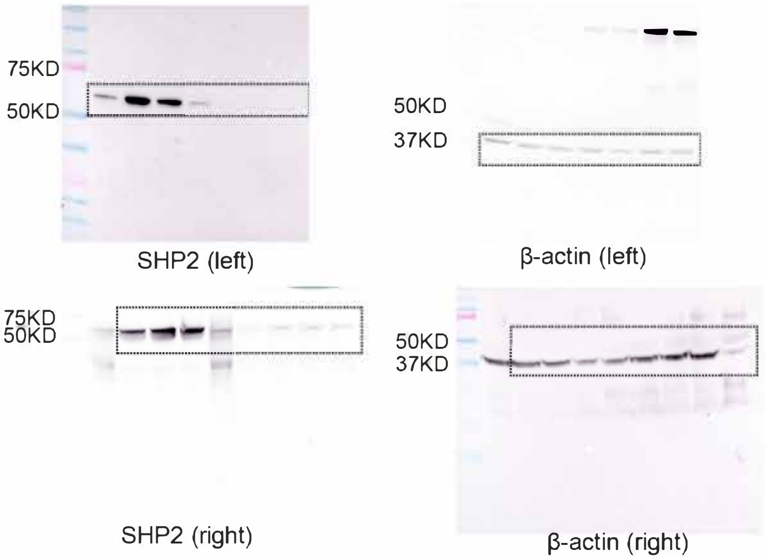

Panel H

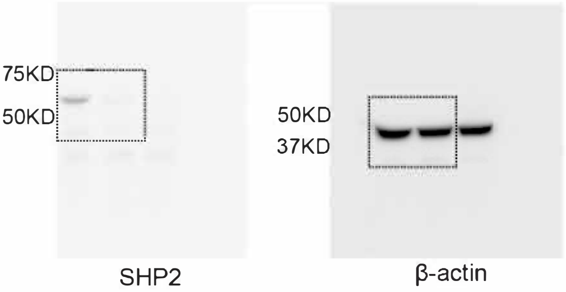

Panel Q

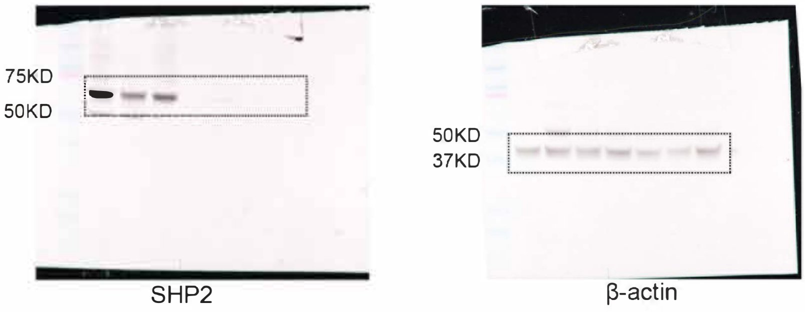

Figure 2

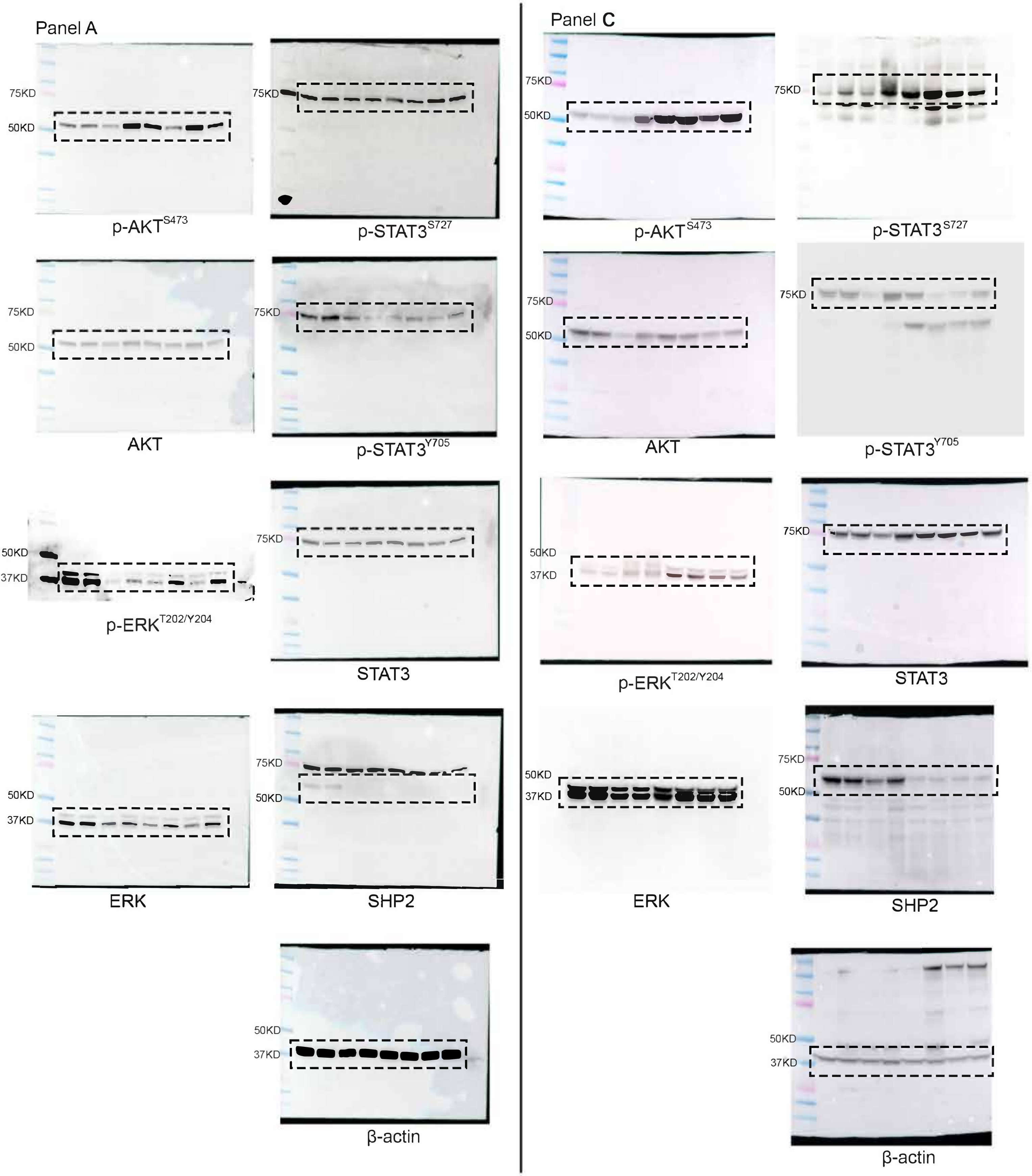

Panel A
75KD
50KD
p-AKT S473
75KD
p-STAT3 S727
75KD
50KD
AKT
75KD
p-STAT3 Y705
50KD
37KD
p-ERK T202/Y204
75KD
STAT3
50KD
37KD
ERK
75KD
50KD
SHP2
50KD
37KD
β-actin
Panel C
75KD
50KD
p-AKT S473
75KD
p-STAT3 S727
75KD
50KD
AKT
75KD
p-STAT3 Y705
50KD
37KD
p-ERK T202/Y204
75KD
STAT3
50KD
37KD
ERK
75KD
50KD
SHP2
50KD
37KD
β-actin

Figure 3

Panel C

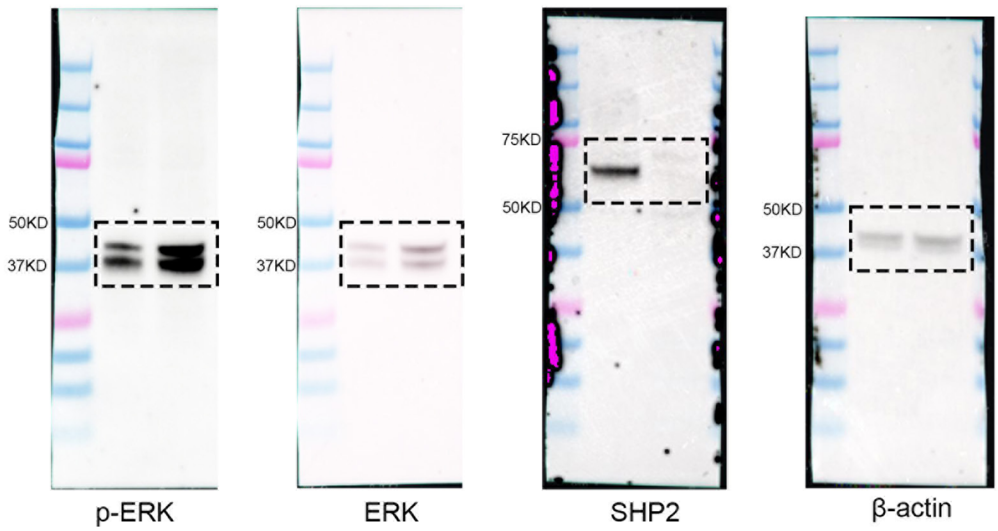

Supplemental Figure 1

Panel A

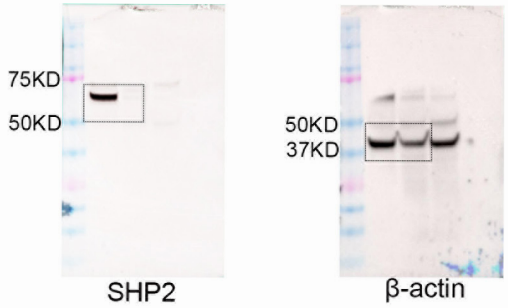

Panel D

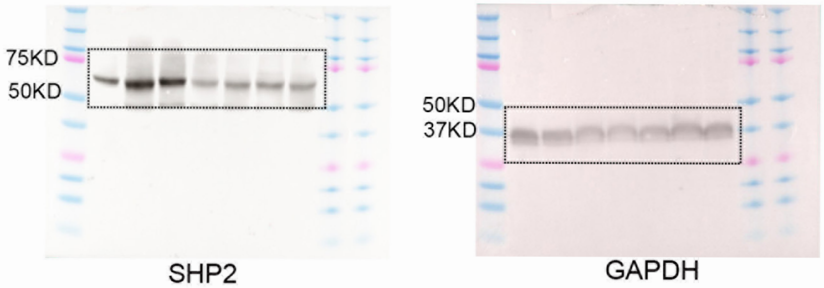

Panel L

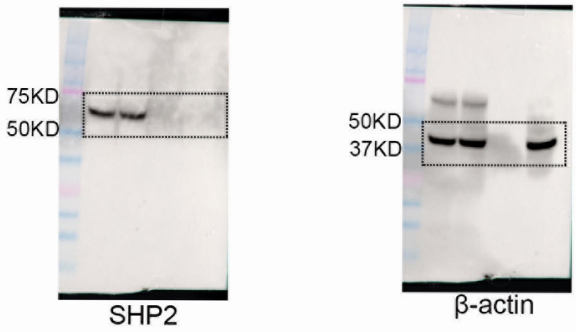

Panel N

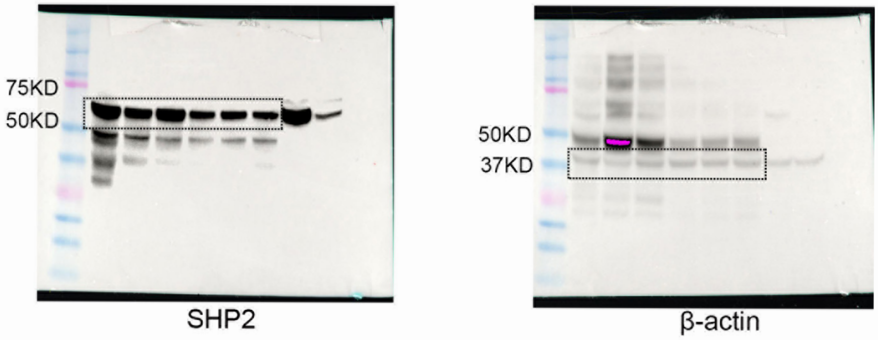

Supplemental Figure 2

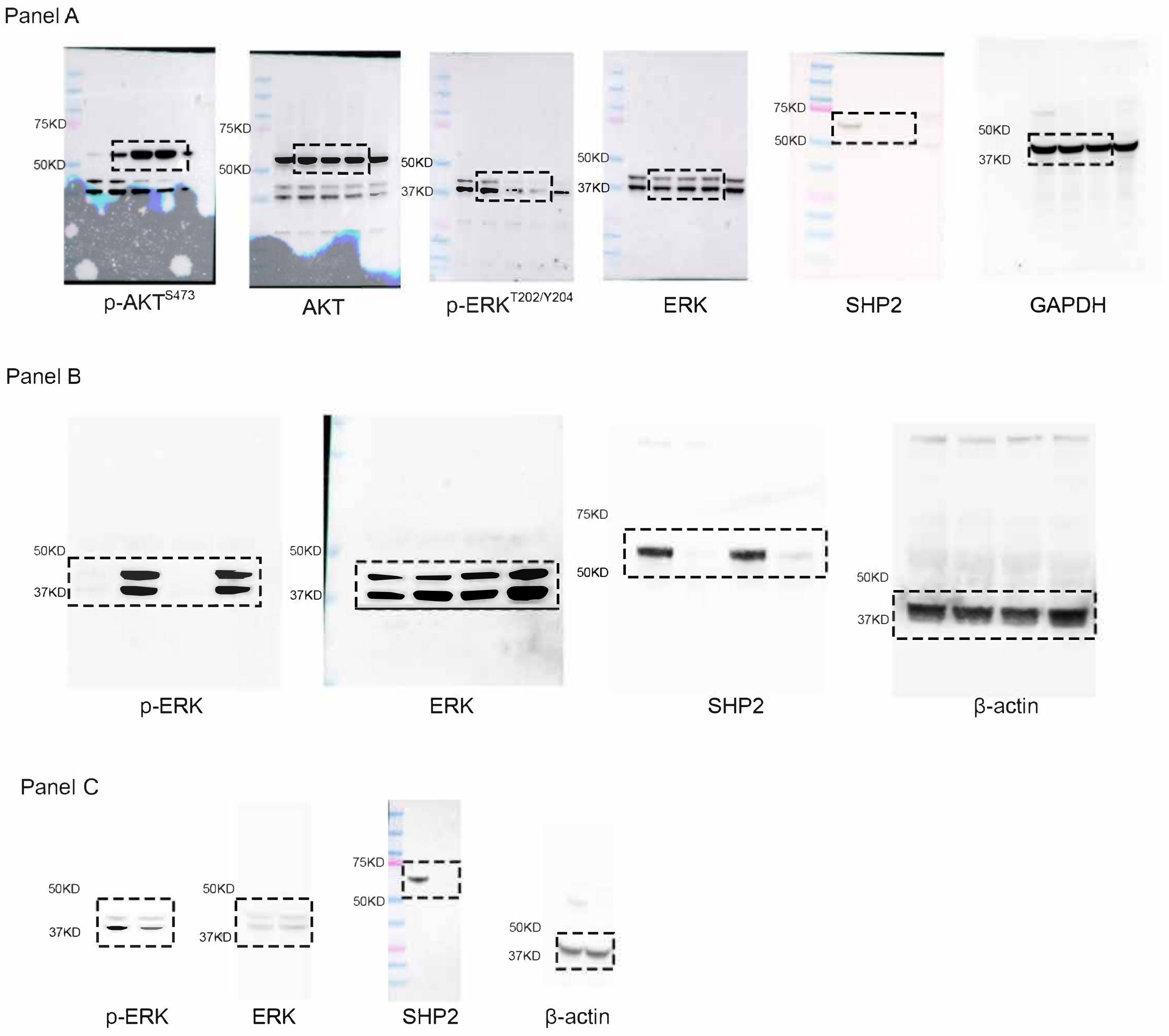

Supplemental Figure 6

Panel B

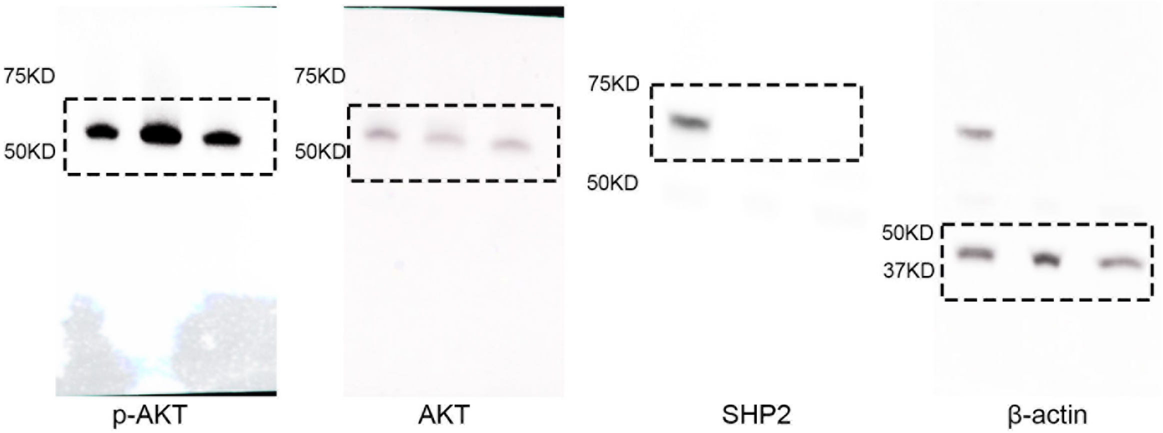

Supplemental Figure 7

Panel C

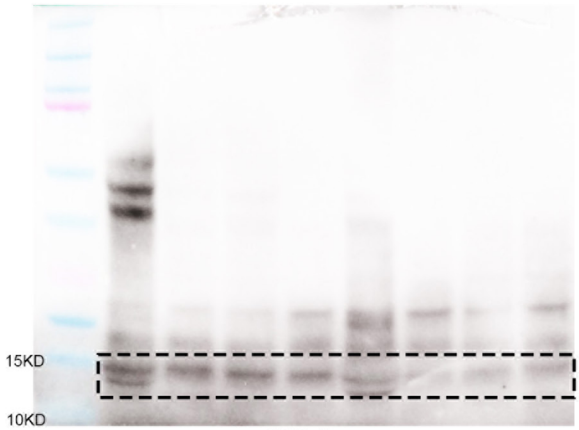

CCL2

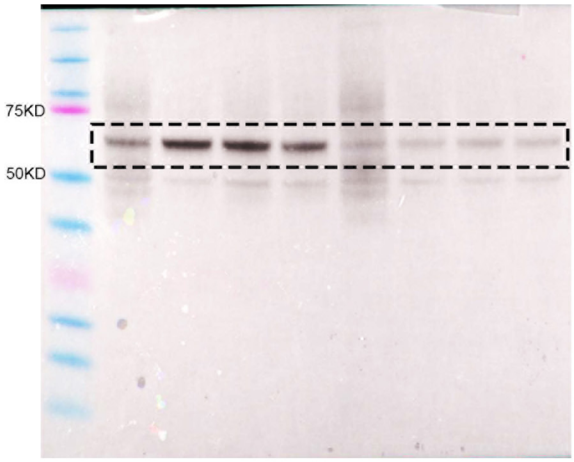

SHP2

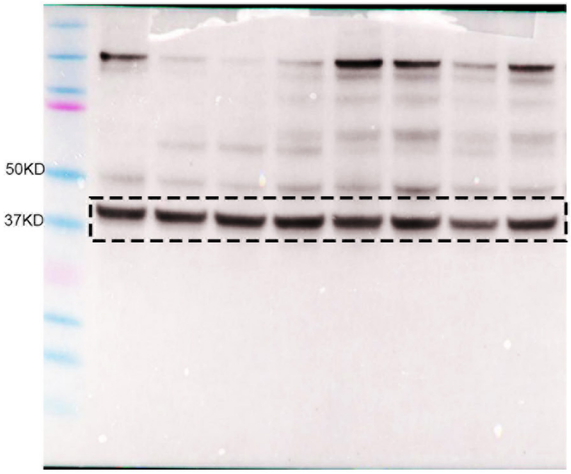

β-actin
